# Impact of climate change and human activity on soil landscapes over the past 12,300 years

**DOI:** 10.1038/s41598-017-18603-4

**Published:** 2018-01-10

**Authors:** Leo Rothacker, Anthony Dosseto, Alexander Francke, Allan R. Chivas, Nathalie Vigier, Anna M. Kotarba-Morley, Davide Menozzi

**Affiliations:** 1 GeoQuEST Research Centre, School of Earth and Environmental Sciences, University of Wollongong, Wollongong, NSW 2522 Australia; 2 Wollongong Isotope Geochronology Laboratory, School of Earth and Environmental Sciences, University of Wollongong, Wollongong, NSW 2522 Australia; 3 University of Cologne, Institute for Geology and Mineralogy, Cologne, D-50674 Germany; 4 Department of Earth Sciences, University of Adelaide, Adelaide, SA 5005 Australia; 5 Laboratoire d’Océanographie de Villefranche (LOV-OOV), CNRS, UPMC, 06230 Villefranche sur Mer, France; 6 Centre for Archaeological Science, School of Earth and Environmental Sciences, University of Wollongong, Wollongong, NSW 2522 Australia

## Abstract

Soils are key to ecosystems and human societies, and their critical importance requires a better understanding of how they evolve through time. However, identifying the role of natural climate change versus human activity (e.g. agriculture) on soil evolution is difficult. Here we show that for most of the past 12,300 years soil erosion and development were impacted differently by natural climate variability, as recorded by sediments deposited in Lake Dojran (Macedonia/Greece): short-lived ( < 1,000 years) climatic shifts had no effect on soil development but impacted soil erosion. This decoupling disappeared between 3,500 and 3,100 years ago, when the sedimentary record suggests an unprecedented erosion event associated with the development of agriculture in the region. Our results show unambiguously how differently soils evolved under natural climate variability (between 12,300 and 3,500 years ago) and later in response to intensifying human impact. The transition from *natural* to *anthropogenic* landscape started just before, or at, the onset of the Greek ‘Dark Ages’ (~3,200 cal yr BP). This could represent the earliest recorded sign of a negative feedback between civilization and environmental impact, where the development of agriculture impacted soil resources, which in turn resulted in a slowdown of civilization expansion.

## Introduction

Soil systems are key components of a broad range of ecosystems. Their development via chemical weathering and mineral dissolution consumes carbon dioxide, and thus plays a substantial role in regulating the carbon cycle and Earth’s climate^1–3^. Erosion plays a major role in chemical weathering, because it exposes unweathered minerals and thus promotes mineral dissolution and soil development. However, accelerated erosion due to deforestation or agricultural practices may inhibit this development, consequently limiting our soil resources in the future^4^. Our understanding of the evolution of soil systems is underpinned by our ability to ascertain how soil erosion and development respond to climatic variability and human activity. It is well known that soil erosion can respond rapidly to changes in rainfall and vegetation cover (anthropogenic or not)^5^. However, much less is known about the variability of soil development over time^6–14^.

In this study, we investigated the relative impact of climate variability and human activity on soil systems during the Late Glacial and Holocene in south-eastern Europe. To achieve this, we used uranium and lithium isotopes in lacustrine sedimentary deposits as proxies for soil erosion and development, respectively. During chemical weathering, ^234^U is preferentially lost from minerals compared to ^238^U, as a result of recoil and preferential leaching^15^. This depletion continues over time since the onset of weathering (*weathering age*;^16^), resulting in decreasing δ^234^U values (δ^234^U = (λ_234_.N_234_/λ_238_.N_238_ − 1) × 1000; unit: parts per thousand, ‰. λ and N are the decay constant (in yr^−1^) and number of atoms for each isotope, respectively; and subscripts 234 and 238 refer to ^234^U and ^238^U, respectively). In a weathering profile, δ^234^U values are expected to decrease with decreasing depth as the weathering age increases (see Supplementary Material for more details). Consequently, if erosion on hillslopes is shallow (e.g. sheet wash), sediments should exhibit negative δ^234^U. Conversely, if deeper erosion (gully, bank, mass wasting) dominates, sediments should be characterized by higher δ^234^U values (Supplementary Figs S8 and S9). Lithium isotopes (^7^Li and ^6^Li) fractionate during clay formation, whereby ^6^Li is preferentially enriched in clay minerals compared to ^7^Li^17,18^. The lithium isotope composition of weathering products, such as clays, therefore reflects weathering conditions shortly before the time of deposition. Clays usually have a δ^7^Li lower than parent material, whereby δ^7^Li = (^7^Li/^6^Li_sample_/^7^Li/^6^Li_L-SVEC_ − 1) × 1000 (unit: parts per thousand, ‰). Experiments have shown that rock or mineral dissolution does not fractionate Li isotopes^19^. In weathering profiles where primary minerals are continuously weathered into clays, soils are increasingly enriched in ^6^Li, preferentially retained in clays over ^7^Li. Thus, decreasing δ^7^Li values reflect increasing clay formation, used as a proxy for the extent of soil development (Supplementary Fig. S7)^20^. Note that unlike U isotopes, Li isotope composition is not expected to vary with soil depth^20,21^,because clay formation occurs pervasively through the weathering profile and is sensitive to hydrological conditions such as the depth of the water table^20^. Recently, Dellinger *et al*.^22^ have shown that the Li isotope composition of river sediments inversely correlates with weathering intensity. This suggests that the δ^7^Li of sediments records the conditions of soil development at the catchment scale. Thus, in this context, the measurement of Li and U isotopes in sedimentary archives such as lake sediments should allow us to determine how soil erosion and development have responded to climatic and/or anthropogenic perturbations over time.

Both Li and U isotope proxies were applied to a sediment core from Lake Dojran at the Macedonian/Greek border. The core is 7 m long and provides a record of paleo-environmental change of the past 12,500 years, at the transition from the cold and arid Younger Dryas into the overall warmer and more humid Holocene^23^. Sediments deposited in this lake are derived from a relatively small watershed (275 km^2^), underlain predominantly by silicate igneous and metamorphic lithologies. The lack of alluvial storage suggests a short sediment transfer time from hillslopes into the lake. Thus, no significant time lag between production of the isotopic signal in soils and deposition in the lake is expected. Furthermore, hydrodynamic sorting is also expected to be minimal. A total of 26 samples were taken from the core and another 11 sediment samples were collected from modern streams draining into Lake Dojran. Additionally, 11 bedrock samples were collected from the catchment. Mineral concentrations were determined, and U and Li isotopes analyzed on size fractions <63 µm at the Wollongong Isotope Geochronology Laboratory, University of Wollongong.

**Figure 1:**
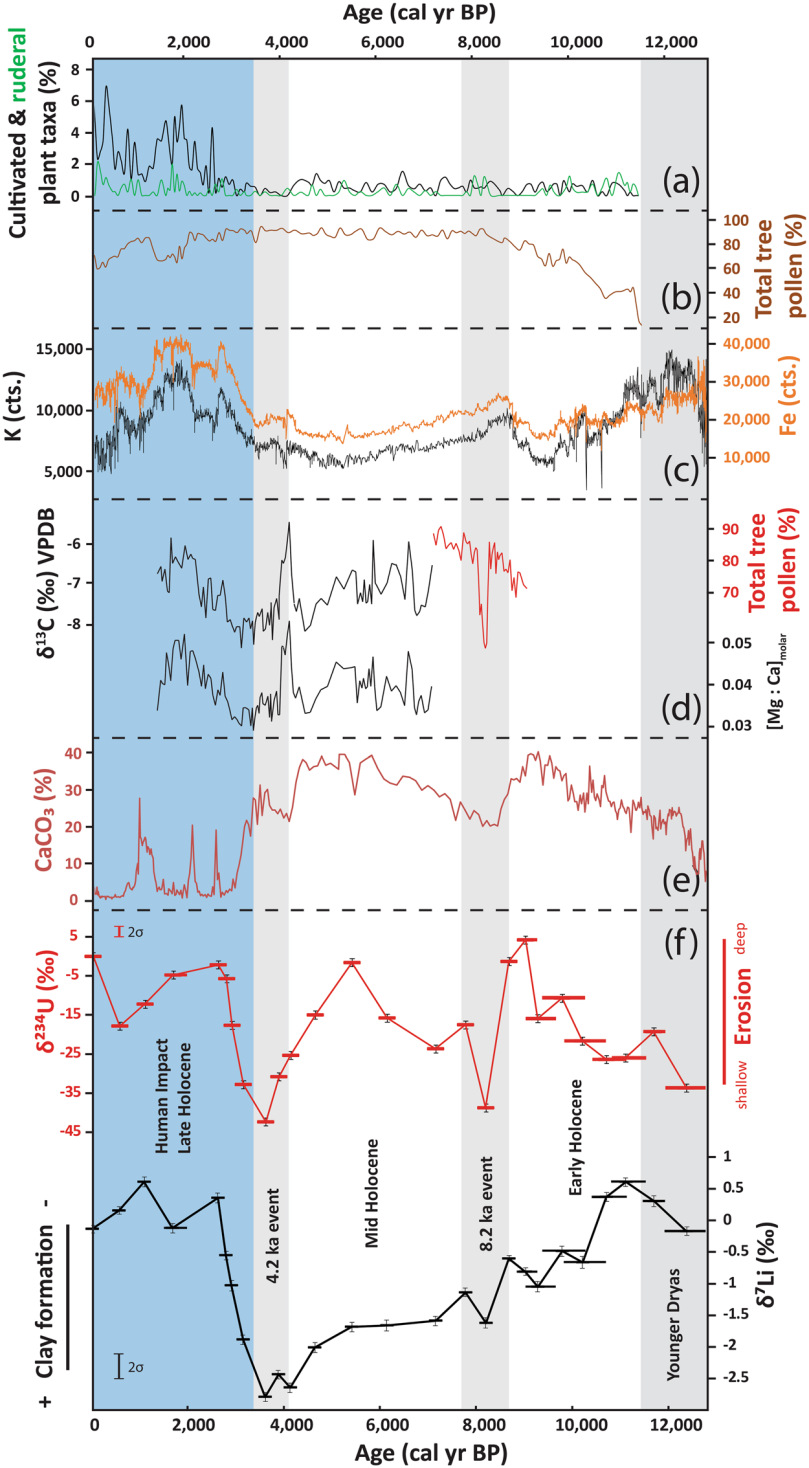
(**a**) Cultivated and ruderal plant taxa in % in Lake Dojran sediment succession. Data from Thienemann *et al*.^30^; copyright given to editor. (**b**) Total tree pollen in Lake Dojran sediment succession. Data from Thienemann *et al*.^30^; copyright given to editor. (**c**) K and Fe concentrations acquired by X-ray Fluorescence (XRF) scan using an ITRAX core scanner (Cox Analytical Systems, Sweden). Measured counts are a semi-quantitative estimate of the relative concentration. Data from Francke *et al*.^23^; copyright is licensed under the Creative Commons Attribution 3.0 License (**d**) Paleo-climate proxies illustrating short-lived cold/dry events at 8.2 and 4.2 kyr BP: pollen data from N Greece (red curve^28^), carbon isotope values (δ^13^C) (black curve) and Mg/Ca ratios (black curve) in a flowstone from N Italy^27^. (**e**) Calcium carbonate (CaCO_3_) concentrations in the same core studied for U and Li isotopes. Data from Francke *et al*.^23^; copyright is licensed under the Creative Commons Attribution 3.0 License. CaCO_3_ concentrations show lake productivity, where high concentrations indicate warm temperatures and low concentrations colder temperatures. (**f**) Lithium and uranium isotope compositions of Holocene core sediment at Lake Dojran. Error bars are 2 standard error for both lithium and uranium data. External reproducibility for both δ^234^U and δ^7^Li is displayed on the left side of the diagram. Error bars for deposition ages are displayed by the horizontal size of the symbol. Grey bands illustrate relatively cool and arid phases, while white bands show warm and wet phases^27–29^. The blue band shows the proposed period of anthropogenic overprint^23,26,45^.

δ^234^U values range from −42.3 ± 1.7‰ to 3.7 ± 1.7‰ with values increasing between 12,300 and 9,000 cal yr BP, followed by a rapid decrease to −39‰ at 8,100 cal yr BP (Fig. 1f). This decline is followed by a return to higher values until 5,300 cal yr BP when another negative trend occurs, reaching a minimum at 3,500 cal yr BP. Between 3,500 and 2,700 cal yr BP, values start increasing rapidly to culminate at 2,500 cal yr BP and then remain high throughout the Late Holocene except for a small negative excursion at 500 cal yr BP. Lithium isotope compositions decrease almost continuously between 12,300 and 3,500 cal yr BP, with δ^7^Li values varying between −2.8 ± 0.4‰ to 0.6 ± 0.4‰. As with U isotope variations, δ^7^Li values increase sharply between 3,500 and 2,500 cal yr BP and remain high for the remainder of the Late Holocene (Fig. 1f).

Low δ^234^U values are observed during the Younger Dryas (12,300–11,700 cal yr BP) and at around 8,100 and 4,000 cal yr BP. On the other hand, δ^234^U displays high values during the Early Holocene (11,700–8,200 cal yr BP), Mid Holocene (4,200–7,800 cal yr BP) and Late Holocene (2,500 cal yr BP – present). This pattern co-varies with climate variability in the Eastern Mediterranean Region over the past 12,300 yr. Several archives show overall higher temperatures and humidity during the Holocene compared to the Late Glacial period, but with short-lived cool/dry events at 8,200 and 4,200 cal yr BP^24–28^. This is consistent with variations in CaCO_3_ concentrations in Lake Dojran sediments^23^, used as a proxy for primary productivity (Fig. 1e). High calcium carbonate concentrations during the Early- to Mid Holocene between 11,700 and 2,500 cal yr BP indicate warm spring and summer temperatures, whereas low calcite concentrations during the Younger Dryas (13,000 and 11,700 cal yr BP) are consistent with a low primary productivity and low spring and summer temperatures. The low calcite concentrations during the Late Holocene (2,500 cal yr BP and present) are mainly a result of dilution with clastic matter, most likely caused by an increase in clastic sediment flux from the catchment. Two excursions towards low calcite concentration centred at 8,200 and 4,000 cal yr BP imply that the lake productivity decreased abruptly over a period of several hundred years. These two cold phases are commonly referred to as the *8*.*2* and *4*.*2 ka events*, which lasted for approximately 400 years^27–29^, and are associated with dry conditions at Lake Dojran^23^, although not captured by the local pollen record^30^. Independent climate data confirm the presence of these two cool and arid phases in the Mediterranean region: tree pollen data obtained from northern Greece show an abrupt decline around 8,200 cal yr BP (Fig. 1d)^28^. Flowstones from western Italy show a positive excursion of δ^13^C and Mg:Ca ratios at 4,000 cal yr BP (Fig. 1d), which is indicative of drier conditions^27^.

Low δ^234^U values during the two cold and dry periods, around 8,200 and 4,000 cal yr BP, are interpreted as reflecting shallow erosion of top soil horizons due to a decrease in annual rainfall. This contrasts with higher values during warmer and wetter periods (Early-, Mid Holocene), when higher rainfall could have resulted in the mobilization of deeper soil layers via gully or stream bank erosion. These results indicate that soil erosion is sensitive to abrupt climatic shifts and may respond on a relatively short timescale of 500–1,000 years.

By contrast, Li isotopes show a gradual decrease from the termination of the Younger Dryas until ~3,500 cal yr BP inclusive. This suggests a continuous production of neo-formed clays illustrating increasingly more developed soils. In this case, it seems that soil development was insensitive to shorter climatic shifts (at 8,200 and 4,200 cal yr BP). Instead, it responded steadily to the termination of the Younger Dryas, during which dry and cold conditions persisted for 1,200 years^31^. These results suggest that while soil erosion is sensitive to shorter climatic variations and responds over only a few hundreds of years, soil development requires longer-term climate shifts to be affected. Interestingly, even in periods where erosion was mobilizing deeper soil material (high δ^234^U), the material exported still exhibited evidence for significant clay abundance in the deposited sediments (low δ^7^Li). This suggests that erosion was not deep enough to ‘reset’ the landscape and export poorly weathered material (i.e. thin to no soil cover).

While Li and U isotopic records are decoupled for most of the past 12,300 years, both proxies show a sharp increase in δ^7^Li and δ^234^U values starting between 3,500 and 3,100 cal yr BP, and culminating at 2,500 cal yr BP (Fig. 1f). For U isotopes, this suggests a relatively rapid shift to deep sources of sediment. For Li isotopes, the increase in δ^7^Li indicates that sediments delivered to the lake are depleted in weathering products and are instead dominated by primary minerals. This suggests that soil cover was diminished to the extent that sediments delivered to the lake consisted mostly of unweathered minerals derived from the bedrock. This is consistent with a sharp increase in iron and potassium concentrations in the same core from 3,500 to ~2,500 cal yr BP, interpreted as an increase in clastic flux to the lake (Fig. 1c). Furthermore, the sharp decrease in endogenic calcite abundance associated with an increase in primary minerals such as muscovite, biotite and augite also suggests an increase in clastic flux (Supplementary Fig. S12). To verify that the observed Holocene variations in both Li and U isotope proxies are not simply a function of varying sediment provenance, we investigated the present sediment sources of the lake, as well as the surrounding bedrock lithology. This is discussed in detail in the Supplementary Material.

**Figure 2:**
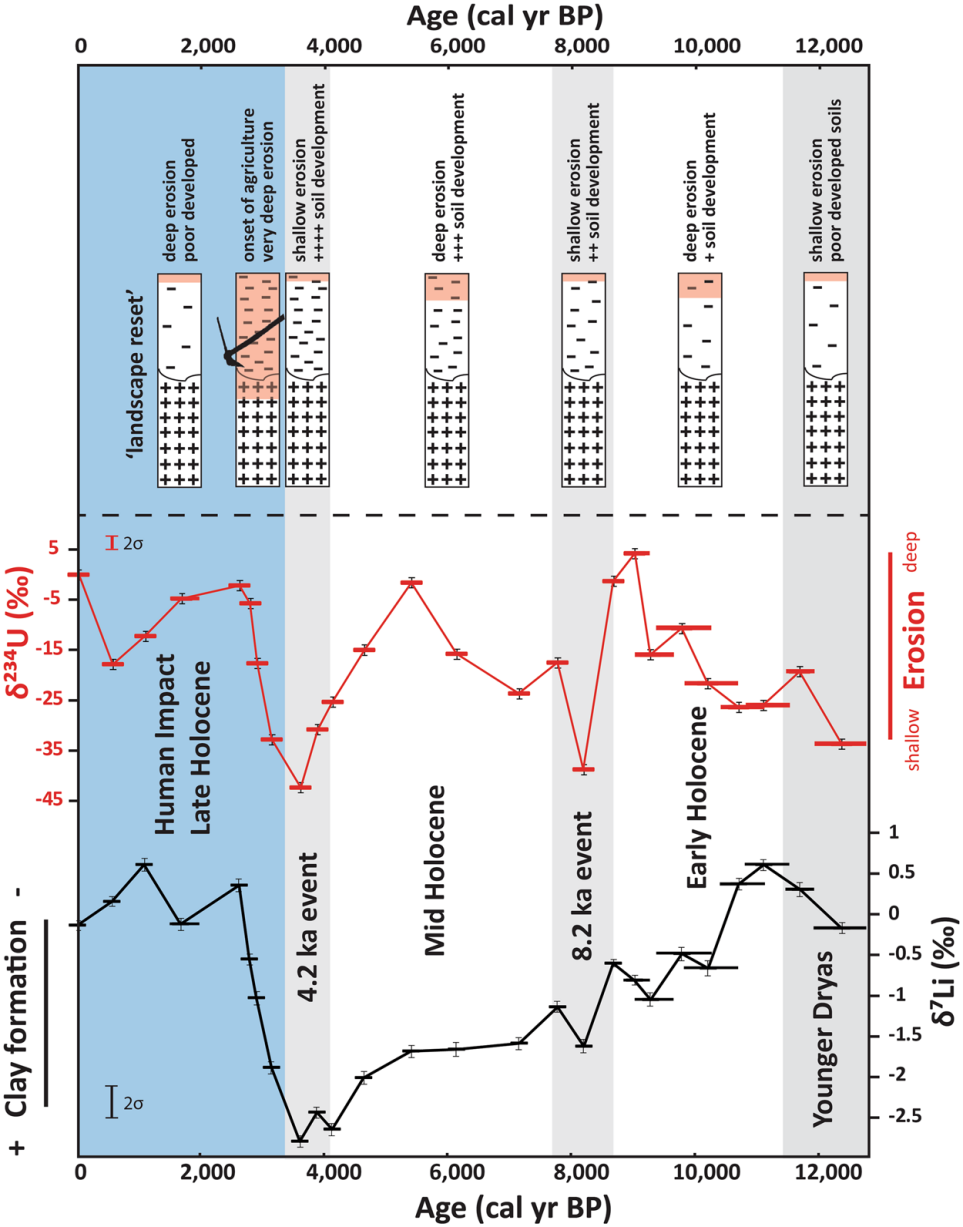
Top panel: Conceptual representation of the evolution of soil profiles at the Lake Dojran catchment throughout the Holocene. Areas highlighted in orange shade represent a schematic erosion depth of individual profiles. Hyphen density conceptualize clay abundances. Soil erosion depth varied over time and responded to short-lived (<1,000 years) climatic events, while clay concentrations increased continuously from 12,000 to 3,500 cal yr BP. Between 3,500 and 3,100 cal yr BP, anthropogenic agricultural practices (as depicted by the plough symbol) caused the mobilization of deeper soil horizons, effectively ‘resetting’ the landscape at ~2,500 cal yr BP to soil formation conditions unprecedented during Holocene. Bottom panel is the same as in Fig. 1f.

Along with geochemical and mineralogical data, both isotope proxies suggest unprecedented environmental changes initiated between 3,500 and 3,100 cal yr BP and peaking at 2,500 cal yr BP. Both lithium and uranium data show that by 2,500 cal yr BP, deep erosion effectively ‘reset’ the landscape, exposing poorly developed soils. Biomarker records suggest a human impact/resettlement at 3,300 cal yr BP^30^. At the same time, an increasing abundance of cultivated and ruderal plant taxa show first signs of agriculture around Lake Dojran (Fig. 1a)^30^. These taxa peaked at 2,600 cal yr BP, implying a strong human impact associated with wide-spread land-use. A significant decrease in total tree pollen at 2,000 cal yr BP then indicates anthropogenic deforestation (Fig. 1b). These observations show that human impact at that time was so profound that it left a distinct trace in the geological record. Since 2,500 cal yr BP and the establishment of an *anthropogenic landscape*, there has been no return to the natural conditions experienced for most of Holocene. While the study area is relatively small (275 km^2^) and observations may be of only local significance, the timing of the establishment of an anthropogenic landscape corresponds to the expansion of urban civilizations and the development of agricultural practices^30^. Studies have shown that pastoralism may also play a role on soil erosion during this time period^12,32^. However, pollen data indicate that the most significant factor to induce the observed erosion event must have been the development of agricultural practices such as plant cultivation, and later widespread deforestation^23,30,33^. The latter could have been triggered by the emergence of regional trade, when large amounts of high-quality Macedonian timber were required for shipbuilding and construction^34^. Additionally, diversifying metal smelting, production of goods, heating and cooking, as well as warfare would have also caused a high demand for wood. Therefore, we suggest that the intensive large-scale land-use, first through plant cultivation at 3,300 cal yr BP and later through deforestation at 2,000 cal yr BP, is the direct cause of the observed erosion event in the Late Holocene (Fig. 2). Interestingly, the erosion event occurred before or at the onset of the Greek ‘Dark Ages’ (~3,200 cal yr BP;^35^). In this period, population declined, agriculture suffered, the metallurgy of bronze and the ability to write were forgotten^35^. Interestingly, the erosion peaked at the same time (2,500 cal yr BP) when the Athenian empire and other southern Greek *poleis* (city states) increased their demand for timber, especially for naval construction. If the erosion event observed at Lake Dojran is of regional significance, the associated stress on natural resources could have triggered or at least contributed to a decline into ‘Dark Ages’. Thus, while humans have impacted their environment since the onset of the Holocene, we show that the expansion of civilization in this region resulted in environmental changes that in turn could have negatively impacted human societies.

## Methods

Sample preparation was undertaken in a Class 10 cleanroom at the Wollongong Isotope Geochronology Laboratory, University of Wollongong. About 10 mg of the <63 µm fraction was dissolved in 48% HF and 65% HNO_3_ at 100 °C for >12 hours. After drying down, samples were redissolved in aqua regia at 130 °C for >12 hr to break down any fluorides. After drying down, samples were redissolved in 1.5 mL 1 M HCl. To separate lithium from the sample matrix, cation exchange chromatography was applied. The method applied in this study was adapted after Balter & Vigier 2014^36^. It is crucial to separate Li from other ions, especially Na, as they may suppress ionization and cause additional isotopic fractionation during analysis^37^. Furthermore, it is essential to recover 100% of Li, otherwise isotopes may fractionate by up to 200‰ during chromatography^17^. For this study, Savillex 30 mL micro columns (6.4 mm internal diameter, 9.6 cm outside diameter, capillary length of 25 cm) were used. Columns were vertically customized to have a capillary length of approximately 12 cm. Biorad AG50W-X8 resin (200–400 mesh) was used as cation exchange medium, with a volume of 3.06 cm^3^ (9.5 cm length). Before sample loading, columns were cleaned using 30 mL of 6 M HCl, rinsed with 2–3 mL 18.2 MΩ water, and conditioned with 8 mL 1 M HCl. The latter acid was titrated to 1 M. Cation exchange columns were calibrated with seawater samples. On two individual columns, it was verified that over 99% of the original Na was removed after two column passes, while maintaining ~100% Li yield (Fig. S5). The lithium elution was dried down and taken up in 0.3 M HNO_3_ for isotopic analysis.

Lithium isotope ratios were measured at the Wollongong Isotope Geochronology Laboratory, University of Wollongong, on a Thermo Neptune Plus^TM^ Multiple Collector Inductively Coupled Plasma Mass Spectrometer (MC ICP-MS). Using wet plasma conditions, a 30 ppb single element lithium tuning solution yielded a typical intensity of 1 V on ^7^Li, while background was of the order 5–30 mV on ^7^Li. A standard bracketing technique was applied^38^ using IRMM16 as primary standard for ^7^Li/^6^Li ratios. Synthetic standards Li7-N and Li6-N^39^ were used to assess accuracy of isotopic ratio determination. Instrument blanks were measured between each standard and sample by introducing 0.3 M HNO_3_. Blank intensities were then subtracted from each isotope. Corrected ^7^Li/^6^Li were converted to δ^7^Li values using L-SVEC as reference^39^. Results for Li7-N and Li6-N were: δ^7^Li = 30.4 ± 0.1‰ (n = 29, 2σ) and −7.8 ± 0.1‰ (n = 31, 2σ), respectively. This is within reported values for each standard: 30.2 ± 0.3‰ (n = 89) and −8.0 ± 0.3‰ (n = 38), respectively^39^ (Table S3, Fig. S6).

To verify the sediment sample dissolution and ion exchange chromatography protocols for Li isotope measurements, a granitic reference material JG-2 was processed along with the samples^40^. The average δ^7^Li value obtained was 0.6 ± 0.1‰ (n = 22, 2σ). Reported δ^7^Li values for JG-2 range from −0.2 to 0.4‰^18,37^ (Table S3, Fig. S6). Total procedure blanks (n = 3) yielded <1 ng of Li, two of them yielding <0.3 ng, which represents 0.06–0.67% of the amount of Li processed for sample analysis (150–500 ng). For all core samples, Li elutions were measured twice yielding isotope compositions within error of each other. Furthermore, for three different samples, two separate aliquots were processed to assess reproducibility. The average 2 standard deviation for these replicate measurements was 0.4‰. Lithium isotope composition of core sediments is shown in Table S4.

For U isotopes, following sample dissolution, a fraction of 1 M HCl solution was dried down and re-dissolved in 1.5 M HNO_3_. Uranium was separated by ion exchange chromatography following the protocol described in Luo *et al*.^41^. Uranium isotopes were analyzed at the Wollongong Isotope Geochronology Laboratory on a Thermo^TM^ Neptune Plus MC ICP-MS. Uranium-234 was collected on a secondary electron multiplier (SEM) and ^235^U and ^238^U on Faraday cups. Measured isotope ratios were corrected for mass bias and Faraday/SEM yield by standard bracketing and using NBL U010 as primary standard^42^. Total procedure blanks (n = 3) yielded <40 pg U, which represents less than 0.01% of the amount of U from the samples (300–600 ng). Accuracy and external reproducibility were assessed using USGS reference material QLO-1, a quartz latite in secular equilibrium^43,44^. Two separate aliquots were processed separately and yielded (^234^U/^238^U) activity ratios of 1.002 ± 0.002 (2σ) and 1.003 ± 0.002 (2σ). Based on these two measurements, we calculated the external reproducibility (relative 2 standard deviation, n = 2) to be 0.17% of measured ^234^U/^238^U activity ratios. Uranium isotope composition of core sediments is shown in Table S4.

Other analytical techniques are presented in the supplementary material.

## Electronic supplementary material

Supplementary material
