# Supplementary material for: Impact of climate change and human activity on soil landscapes over the past 12,300 years
Source: Sci Rep. 2018 Jan 10;8:247. doi: 10.1038/s41598-017-18603-4 (PMC5762867; doi:10.1038/s41598-017-18603-4)
Supplement: Supplementary file 1 — Supplementary material [file 41598_2017_18603_MOESM1_ESM.pdf]

Impact of climate change and human activity on soil landscapes over the past 12,300 years

LEO ROTHACKER[1,2*], ANTHONY DOSSETO[1,2], ALEXANDER FRANCKE[1,2,3], ALLAN R. CHIVAS[1,4], NATHALIE VIGIER[5], ANNA M. KOTARBA-MORLEY[6] AND DAVIDE MENOZZI[1,2]

[1]GeoQuEST Research Centre, School of Earth and Environmental Sciences, University of Wollongong, Wollongong, NSW 2522, Australia

[2]Wollongong Isotope Geochronology Laboratory, School of Earth and Environmental Sciences, University of Wollongong, Wollongong, NSW 2522, Australia

lr623@uowmail.edu.au; tonyd@uow.edu.au; afrancke@uow.edu.au; toschi@uow.edu.au; dm791@uowmail.edu.au

[3]University of Cologne, Institute for Geology and Mineralogy, Cologne, D-50674, Germany

[4]Department of Earth Sciences, University of Adelaide, Adelaide, SA 5005, Australia

[5]Laboratoire d'Océanographie de Villefranche (LOV-OOV), CNRS, UPMC, 06230 Villefranche sur Mer, France

nathalie.vigier@obs-vlfr.fr

[6]Centre for Archaeological Science, School of Earth and Environmental Sciences, University of Wollongong, Wollongong, NSW 2522, Australia

amorley@uow.edu.au

*corresponding author: lr623@uowmail.edu.au

The Supplementary Material includes:

- Study area and sampling strategy
- Lake sediment chronology
- Analytical techniques
- Supporting data
- Lithium isotopes as proxy for soil formation
- Uranium isotopes as proxy for soil erosion
- Discussion of the different hypotheses to explain Li and U isotopic variations
- Tables and figures

Study area and sampling strategy

Lake Dojran is situated on the border between Macedonia and Greece (41°12'N, 22°44'E). Water surface area and average water depths were measured in 2004 at 40 km^2 and 3-4 m, respectively[1]. The catchment area (275 km^2) is drained by small rivers, creeks, and groundwater. The only outflow of Lake Dojran, located in the southern corner of the lake, was canalized in the 1950's. In the eastern part of the catchment, the lithology underlying the drainage area is composed of gneiss, granite, mica schist, amphibolite, Quaternary alluvial sediment, and a small area of volcanic-sedimentary rock at the southern end of the lake (Fig. S1). In the western part, the lithology consists of muscovite gneiss, green schist, gabbro, serpentinite, biotite gneiss, granite, and marble.

Lake Dojran is mostly fed by streams originating in the north-east east (NE-E) part of the catchment and transit through a small alluvial plain before reaching the lake (Fig. S1, S2). Streams in the northern, western and southwestern part of the catchment are ephemeral and have a low sediment transport capacity, therefore contribute little sediment to the lake. The only outlet is at the southeastern end of the lake and used to connect Lake Dojran with the Axios/Vardar River. Today this outlet is located several meters above lake level. Water loss is therefore only through evaporation and possibly groundwater outflow[2].

The study area is climatically influenced by mid-latitude westerlies and the Subtropical High pressure belt. The North Atlantic Oscillation (NAO) modulates winter precipitation and the migration of the Intertropical Convergence Zone (ITCZ) affects dry periods during summer[3]. Precipitation is highest during mild winters (612 mm/yr), and lowest during hot summers. Mean annual air temperature around the lake averaged 14.3°C between 1961 and 2000,

whereas mean monthly summer and winter temperatures were 26.1°C and 3.7°C, respectively[1].

Stream sediments

To study the present weathering environment, sediments from streams draining into Lake Dojran were collected (Fig. S1, S2). Sampling was undertaken in December 2015. Most streams were smaller than 2 m in width, and had little to no water present at the time of sampling (Fig. S3).

Core sediments

The sediment core was drilled in June 2011 using a gravity corer for undisturbed surface sediments and a percussion piston corer for deeper sediment layers. More details can be found in Francke et al. 2013[2]. Core sediments consist predominantly of silt-sized material with varying concentrations of authigenic (endogenic calcite, organic matter, biogenic silica) and clastic matter. Average mean grain size over the whole core varies between 12 and 45 µm. The overall mottled to massive structure of the sediment suggests bioturbation. This is also reflected by the relatively high abundance of ostracods, which indicates that there was sufficient oxygen available for larger benthic organisms[2].

Lake sediment chronology

The previously published age model for the analyzed sediment succession[2] has been re-calculated (Table 1, Fig. S4) using the R64 based software package clam2.2[4] and the IntCal13 calibration curve[5]. This was done in order to obtain an estimate on the uncertainty of the deposition age for individual samples (Table S2). Out of a total of 13 radiocarbon ages, 9 were used for the age depth model interpolation. The 4 excluded samples are considered to be affected by a reservoir and/or hard water effect, are re-deposited, or have been relocated during the core opening[2]. In addition, the minima in $CaCO_3$ at 397 cm sediment depth has been correlated to 8,200 cal yr BP, as similar patterns, associated with the 8.2 cooling event, have also been described at nearby lakes Prespa and Ohrid[6-9].The analyzed lake sediment record covers the past 13,000 years with an average sedimentation rate of ~54 cm/1,000 years[2].

Analytical techniques

Grain size distribution

While grain size distribution on bulk samples was previously measured for the core in Francke et al. 2013[2], it was determined again for the <63 µm fraction of the samples analyzed in our study. Prior to measurements, samples were treated with 30% H_2O_2, 10% HCl, and NaOH to remove organic matter, endogenic calcite and biogenic silica, respectively. Samples were then sieved to 63µm and grain size analyses were carried out on the <63 µm fraction at the University of Cologne using a Beckman LS™ 13 320 particle size analyzer.

Mineralogy

Mineralogical compositions were determined by X-ray diffraction at the University of Wollongong. An aliquot of each lake sediment sample was dried at 50-60°C. The samples were then mounted in aluminium holders and placed in a Phillips 1130/90 diffractometer with Spellman DF3 generator set to 1 kW. The 1 kW energy is achieved by setting the diffractometer to 35 kV and 28.8 mA. Samples were analyzed between 4 and 70° 2-theta at 2° per minute with a step size of 0.02. Traces were produced through a GBC 122 control system and analyzed using Traces, UPDSM and SIROQUANT softwares.

Lithium and uranium isotopes

Further sample preparation was undertaken in a Class 10 cleanroom at the Wollongong Isotope Geochronology Laboratory, University of Wollongong. About 10 mg of the <63 µm fraction was dissolved in 48% HF and 65% HNO_3 at 100°C for >12 hours. After drying down, samples were redissolved in aqua regia at 130°C for >12 hr to break down any fluorides. After drying down, samples were redissolved in 1.5 mL 1M HCl. To separate lithium from the sample matrix, cation exchange chromatography was applied. The method applied in this study was adapted after[10]. It is crucial to separate Li from other ions, especially Na, as they may suppress ionization and cause additional isotopic fractionation during analysis[11]. Furthermore, it is essential to recover 100% of Li, otherwise lithium isotopes may fractionate by up to 200‰ during chromatography[12]. For this study, Savillex 30 mL micro columns (6.4 mm internal diameter, 9.6 cm outside diameter, capillary length of 25 cm) were used. Columns were vertically customized to have a capillary length of approximately

12 cm. Biorad AG50W-X8 resin (200-400 mesh) was used as cation exchange medium, with a volume of 3.06 cm^3 (9.5 cm length). Before sample loading, columns were cleaned using 30 mL of 6M HCl, rinsed with 2-3 mL 18.2 MΩ water, and conditioned with 8 mL 1M HCl. The latter acid was titrated to 1 molar. Cation exchange columns were calibrated with seawater samples. On two individual columns, it was verified that over 99% of the original Na was removed after two column passes, while maintaining ~ 100% Li yield (Figures S5). The lithium elution was dried down and taken up in 0.3M HNO_3 for isotopic analysis.

Lithium isotope ratios were measured at the Wollongong Isotope Geochronology Laboratory, University of Wollongong, on a Thermo Neptune Plus™ Multiple Collector Inductively Coupled Plasma Mass Spectrometer (MC ICP-MS). Using wet plasma conditions, a 30 ppb single element lithium tuning solution yielded a typical intensity of 1 V on ^7Li, while background was of the order 5-30 mV on ^7Li. A standard bracketing technique was applied[13] using IRMM16 as primary standard for $^7Li/^6Li$ ratios. Synthetic standards Li7-N and Li6-N[14] were used to assess accuracy of isotopic ratio determination. Instrument blanks were measured between each standard and sample by introducing 0.3M HNO_3. Blank intensities were then subtracted from each isotope. Corrected $^7Li/^6Li$ were converted to δ^7Li values using L-SVEC as reference[14]. Results for Li7-N and Li6-N were: δ^7Li = 30.4 ± 0.1 ‰ (n=29, 2σ) and -7.8 ± 0.1 ‰ (n=31, 2σ), respectively. This is within reported values for each standard: 30.2 ± 0.3 ‰ (n=89) and -8.0 ± 0.3 ‰ (n=38), respectively[14] (Table S3, Fig. S6).

To verify the sediment sample dissolution and ion exchange chromatography protocols for lithium isotope measurements, a granitic geochemical reference material JG-2[15] was processed along with the samples. The average δ^7Li value obtained is 0.55 ± 0.13 ‰ (n=22, 2σ). Reported δ^7Li values for JG-2 range from -0.2 to 0.4 ‰[11,16] (Table S3, Fig. S6). Total procedure blanks (n=3) yielded <1 ng of Li, two of them yielding <0.3 ng, which represents 0.06 – 0.67 % of the amount of Li processed for sample analysis (150-500 ng). For all core samples, Li elutions were measured twice yielding isotope compositions within error of each other. Furthermore, for three different samples, two separate aliquots were processed to assess reproducibility. The average 2 standard deviation for these replicate measurements was 0.4 ‰. Lithium isotope composition of core sediments is shown in Table S4.

For uranium isotopes, following sample dissolution, a fraction of 1M HCl solution was dried down and re-dissolved in 1.5M HNO_3. Uranium was separated by ion exchange

chromatography following the protocol described in[17]. Uranium isotopes were analyzed at the Wollongong Isotope Geochronology Laboratory using the same Thermo™ Neptune Plus MC ICP-MS. Uranium-234 was collected on a secondary electron multiplier (SEM) and ^{235}U and ^{238}U on Faraday cups. Measured isotope ratios were corrected for mass bias and Faraday/SEM yield by standard bracketing and using NBL U010 as primary standard[18]. Total procedure blanks (n=3) yielded <40 pg U, which represents less than 0.01% of the amount of U from the samples (300-600 ng). Accuracy and external reproducibility were assessed using USGS reference material QLO-1, a quartz latite in secular equilibrium[19,20]. Two separate aliquots were processed separately and yielded ($^{234}U/^{238}U$) activity ratios of 1.002 ± 0.002 (2σ) and 1.003 ± 0.002 (2σ). Based on these two measurements, we calculated the external reproducibility (relative 2 standard deviation, n=2) to be 0.17% of measured $^{234}U/^{238}U$ activity ratios. Lithium isotope composition of core sediments is shown in Table S4.

A third fraction after sample dissolution was analyzed for major elements using a Quadruple Inductively Coupled Plasma Mass Spectrometer (Q ICP-MS) at the Wollongong Isotope Geochronology Laboratory. Element concentrations were determined using a range (n=8) of calibration standards covering concentrations between 0.05 ppb and 200 ppb. Concentrations of all measured samples fell within this range. The blank intensity of a 0.3M HNO_3 was subtracted from each sample. For quality control, a standard solution with a known element concentration was analyzed after every 10 samples. To assess reproducibility, two separate aliquots were measured. The relative two standard error of these measurements were 15% and 14% for K and Ti, respectively.

Lithium isotopes in lake sediments as a proxy for soil development

Lithium isotopes (^7Li, ^6Li) fractionate during secondary mineral formation, where the lighter isotope ^6Li is preferentially retained in secondary phases such as clay minerals. Iron oxides show similar behaviour retaining ^6Li, however lithium is generally depleted and therefore the lithium contribution of Fe oxides to the overall lithium pool in the analyzed sediment is negligible[21]. Compared to primary minerals, clays are enriched in lithium and display relatively low lithium isotope ratios[16]. Sediments analyzed in this study consist of a mixture between secondary phases and primary minerals. As soils develop, more clays, enriched in lithium with relatively low δ^7Li, are expected to be present in the sediment mixture. In turn, this drives the lithium isotope ratio of the sediment to lower values. In a recent study,

Dellinger et al. 2017[22] found a negative relationship between the lithium isotope ratio of fine river sediments and the weathering intensity (ratio between silicate weathering rate and total denudation rate). They show that δ^7Li values of sediments are more negative for high weathering intensities. Therefore, δ^7Li in sedimentary deposits could reflect changes in soil development at a given catchment over time. For large catchments with a mixed lithology, the lithium isotope composition of sediments may vary depending on the δ^7Li of the source rock[22]. In our study, considering the small catchment area of Lake Dojran (275 km^2) and the limited sediment pathways into the lake (Fig. S2), we assume that bedrock variability does not have a major influence on δ^7Li of the lake sediments. Thus, in this context, δ^7Li of the sedimentary record can be used to reconstruct paleo-variations in soil development at the time of deposition. Our conceptual illustration of the lithium isotope behaviour during soil development is shown in Fig. S7. Alternative hypotheses to explain variations in δ^7Li in sediments are: (1) sediment provenance (2) mineralogical sorting and (3) post depositional processes. These hypotheses are discussed below.

Uranium isotopes in lake sediments as proxy for soil erosion

In fine-grained soil/sediment, ^{234}U is lost at the mineral surface such that ^{234}U/^{238}U decreases over time. In weathering profiles, as the weathering front moves downward, soil age is expected to increase with decreasing depth and as a result δ^{234}U becomes more negative (Fig. S8). Decreasing δ^{234}U values with decreasing soil depth have been observed in the top meter of weathering profiles developed in temperate climate over a range of lithologies, from shale to granodiorite (Fig. S9). During shallow erosion, as only the top soil horizon is being mobilized, sediments are expected to display negative δ^{234}U values. In contrast, if erosion mobilizes deeper parts of the soil profile, the resulting sediments are to display higher δ^{234}U values (Fig. S8). Thus, in sedimentary records (e.g. lakes), the δ^{234}U of the sediment could be used as a proxy for changes in soil erosion at the catchment scale. However, a difference in sediment sources, mineralogical sorting and post depositional processes could impact/control the δ^{234}U record. These alternative scenarios are discussed below.

Sediment sources in the Lake Dojran catchment

In this section, we test whether variations in U and Li isotopic compositions in lake sediments could reflect changes in sediment provenance. To test this hypothesis, sediments were collected from streams feeding into Lake Dojran and analysed for Li isotopes; bedrock samples were collected from outcrops across the catchment and analysed for U isotopes.

Stream sediments δ^7Li values range from -4.8 to +0.8 ‰ (Table S5). Streams with the most negative values are located in the western part of the catchment. The Li isotope composition of lake sediments could potentially be controlled by changes in sediment provenance if, for instance, there was an increase contribution from western streams. However, the drainage area and channel length of these streams are small compared to those in the E-NE. Furthermore, western streams have narrow and shallow channels (<1 m^2) indicating a small sediment transport capacity. Consequently, these streams should have a very small contribution to the sediment budget of the lake. Sediments are more likely to be derived from eastern and north-eastern streams. There, δ^7Li compositions are close to 0 ‰, similarly to the composition of the youngest lake sediments. Thus, sediment provenance alone cannot account for variations in δ^7Li compositions in lake sediments. Similarly, a change in the location of human activity around 3,500 cal yr BP from the western to the eastern parts of the catchment could not account for the increase in δ^7Li compositions in lake sediments, because of the minor role of western streams on the lake sediment budget.

Eleven bedrock samples were collected from outcrops around the catchment and measured for U isotope ratios (Table S10, Fig. S16). Three samples show δ^{234}U compositions lower than the lowest values observed in lake sediments. Thus, it could be tempting to explain negative δ^{234}U excursions in the lake sediment record by a greater contribution of sediments from areas draining these lithologies (Fig. S16). However, the morphology of the streams draining these areas suggests that they would deliver only a small flux of sediments to the lake. Furthermore, because streams from these areas show overall low δ^7Li values, if these regions dominated for some reason the sediment budget, this would possibly result in low δ^7Li in lake sediments at the same time as low δ^{234}U are observed. This is not the case. Nevertheless, the areas delimited in Fig. S16 are restricted by the small number of bedrock

samples, which does not allow a rigorous assessment of the variability of U isotope compositions to be conducted.

Grain size distribution and mineralogical sorting

The mean grain size of the <63 µm fraction in the Lake Dojran sediment succession varies over the Holocene from 15 to 24 µm. From 12,300 to 6,000 cal yr BP, the mean grain size is variable and shows two minima at 11,600 and 9,200 cal yr BP. From 6,000 to 2,700 cal yr BP, values decrease followed by a gradual increase after 2,700 cal yr BP (Table S6, Fig. S11). Grain size distribution at the coring location is mainly affected by the size of Lake Dojran, i.e. by a combination of wave action, lake-internal current systems, and the shoreline distance.

Lithium isotope data shows no correlation with mean grain size of the sediment core succession (not shown). Similarly, uranium isotope data does not significantly correlate with mean grain size (R^2 = 0.0094) (Fig. S11). Therefore, we can conclude that hydrodynamic sorting does not play a role in Li and U isotope compositions of lake sediments.

Isotopic variations could also potentially be explained by variable mineral sorting over time, although small lake sediments are considerably less affected by these processes compared to deltaic sediments of large river basin. Major minerals in the core sediments are calcite, quartz, muscovite, biotite and chlorite (Table S6 and Fig. S12). Between 12,000 and 3,000 cal yr BP, while calcite was a major phase, it almost completely disappeared after 3,000 cal yr BP. This is mainly the result of mutual dilution with clastic matter, caused by an increase in sediment flux. Other mineral phases show no obvious systematic changes. Considering the mineralogical composition of Lake Dojran sediments (Fig. S12) biotite is likely to dominate the Li and U budgets. No relationship is observed between $\delta^{234}U$ and biotite (or any other mineral) content (not shown), suggesting that variable mineral content is not responsible for the observed $\delta^{234}U$ variations. Lithium isotope compositions show a broad positive relationship with biotite content (Fig. S13). This could suggest that higher $\delta^{7}Li$ values are explained by the higher abundance of biotite. This is confirmed by analysis of handpicked biotite grains from stream sediments, which display an average $\delta^{7}Li$ of ~2.2 ± 0.2‰ (Table S8). The role of biotite on the Li isotope composition of sediments is in agreement with Li isotopes recording the extent of soil development: poor soil development yields sediments enriched in primary minerals such as biotite, which then dominates the Li isotope

composition of the sediment. More thorough soil development yields sediments enriched in secondary minerals such as clays, which dominate the δ^7Li of sediments with low values. Another possible contribution to the observed δ^7Li is the formation of Fe-Mn oxyhydroxides. Similarly to clay minerals, Fe-Mn oxyhydroxides formed during weathering at low temperatures preferentially take up ^{6}Li and an enrichment in Fe-Mn oxyhydroxides could therefore drive the Li isotope composition of a sediment mixture towards low δ^7Li values[23]. Hence, negative δ^7Li of sediments could reflect a combination of clay neo-formation and Fe-Mn oxide precipitation, both indicative of increasing soil formation. When comparing the lithium isotope data with other weathering indices such as K/Ti, we observe a relatively good correlation (Table S6, Fig. S14 & S15). Potassium is mobile during weathering, while titanium is in most cases immobile. In a sediment mixture of silt and clay, K/Ti ratios may be interpreted as i) reflecting chemical weathering reactions, where K is leached from soils or ii) being controlled by potassium-rich phases such as biotite. In both scenarios, low K/Ti ratios reflect increased chemical weathering and soil development. The sample at ~3,500 cal yr BP that shows an increase in K/Ti is most likely an outlier as it neither follows the general trend of K/Ti over time, nor does it correlate with other proxies such as Li isotopes.

Post-depositional processes

While it is possible that Li could be exchanged between sediments and lake water following deposition, it has been shown that clays release an insignificant amount of Li back into solution, which suggests a one-way transfer of Li from solution to clay[12]. Furthermore, given the relatively low Li concentration of natural waters (in the range of parts per billion; e.g.[24]), compared to clays (in the range of parts per million; e.g.[25]), post-depositional exchange of Li between clays and water is unlikely to impact the overall Li isotopic composition of sediments. Experiments have shown that for smectite, the exchangeable fraction of Li is negligible compared to structural lithium[26]. Nevertheless, in order to ensure that the exchange between clay and water represents an insignificant proportion of the sediment Li isotopic budget, we measured the Li isotope ratio of a clay sample for which two aliquots were prepared differently: one aliquot had the exchangeable Li was removed prior to analysis, while the exchangeable Li was not removed in the other aliquot. In each case, no pre-treatment for carbonate was performed as this mineral was not detected in the sample. Organic matter was removed using H_2O_2 before the experiment. The first aliquot was

treated for one hour at room temperature with 8mL 1M NH_4Cl for ~15 mg of sample[27]. The sample was then washed for 5 minutes with 18.2 MΩ water and processed for Li isotope analysis as described above. The aliquot where the exchangeable Li was removed (Clay_1_leached), yielded a δ^7Li value of -0.4 ± 0.2 ‰ (2σ) (Table S9). This is slightly higher than the δ^7Li value measured in the aliquot where the exchangeable Li was not removed (Clay_1): - 1.0 ± 0.2 ‰ (2σ) (Table S9). The experiment was repeated for another sample, and the δ^7Li value of the aliquot where the exchangeable Li was removed (Clay_2_leached), was -0.1 ± 0.2 ‰ (2σ) (Table S9). This is within error compared to the δ^7Li composition of the aliquot were the exchangeable Li was not removed (Clay_2): -0.40 ± 0.3 ‰ (2σ) (Table S9). These results suggest that the exchangeable Li plays a minor role on the sediment Li budget. Thus, post-depositional alteration is unlikely to significantly affect the Li isotope compositions of lake sediments, which is likely to record environmental conditions prior to deposition. Similarly, the pool of exchangeable U is negligible[28] thus any water-sediment interaction post-deposition would be unlikely to affect the δ^{234}U compositions.

The presence of authigenic carbonates within the lake could also contribute to the Li isotopic composition of sediment. They generally form in equilibrium with the lake water, which typically shows relatively positive δ^7Li values. However, carbonates are particularly depleted in Li (Li concentrations typically <1 ppm;[29]), therefore their contribution to the isotopic composition of the sediment is likely to be negligible. Another possible process affecting δ^7Li values post deposition is mineral phase transformation: illitization of smectites. Experiments have shown that lithium isotope fractionation may occur during illitization, where products are isotopically lighter[30]. If this were the case in our study, the δ^7Li of sediments would decline with increasing illitization. So far, post-depositional illitization of smectites has only been observed for lake deposits in highly saline environments[31]. Therefore, considering the low salinity of Lake Dojran waters, this process is unlikely to take place.

References

1. Katsavouni, S. & Petkovski, S. Lake Doiran-An overview of the current situation. *Greek Biotope/Wetland Center (EKBY), Society for the Investigation and Conservation of Biodiversity and the Sustainable Development of Natural Ecosystems (BIOECO)* (2004).
2. Francke, A., Wagner, B., Leng, M. J. & Rethemeyer, J. A Late Glacial to Holocene record of environmental change from Lake Dojran (Macedonia, Greece). *Climate of the Past* **9**, 481-498 (2013).
3. Lionello, P. *et al.* The Mediterranean climate: an overview of the main characteristics and issues. *Developments in earth and environmental sciences* **4**, 1-26 (2006).
4. Blaauw, M. Methods and code for 'classical'age-modelling of radiocarbon sequences. *quaternary geochronology* **5**, 512-518 (2010).
5. Reimer, P. J. *et al.* IntCal13 and Marine13 radiocarbon age calibration curves 0–50,000 years cal BP. *Radiocarbon* **55**, 1869-1887 (2013).
6. Wagner, B. *et al.* A 40,000-year record of environmental change from ancient Lake Ohrid (Albania and Macedonia). *Journal of Paleolimnology* **41**, 407-430 (2009).
7. Wagner, B., Vogel, H., Zanchetta, G. & Sulpizio, R. Environmental change within the Balkan region during the past ca. 50 ka recorded in the sediments from lakes Prespa and Ohrid. *Biogeosciences* **7**, 3187-3198 (2010).
8. Vogel, H., Wagner, B., Zanchetta, G., Sulpizio, R. & Rosén, P. A paleoclimate record with tephrochronological age control for the last glacial-interglacial cycle from Lake Ohrid, Albania and Macedonia. *Journal of Paleolimnology* **44**, 295-310 (2010).
9. Aufgebauer, A. *et al.* Climate and environmental change in the Balkans over the last 17 ka recorded in sediments from Lake Prespa (Albania/FYR of Macedonia/Greece). *Quaternary International* **274**, 122-135 (2012).
10. Balter, V. & Vigier, N. Natural variations of lithium isotopes in a mammalian model. *Metallomics* **6**, 582-586 (2014).
11. James, R. H. & Palmer, M. R. The lithium isotope composition of international rock standards. *Chemical Geology* **166**, 319-326 (2000).
12. Pistiner, J. S. & Henderson, G. M. Lithium-isotope fractionation during continental weathering processes. *Earth and Planetary Science Letters* **214**, 327-339 (2003).
13. Flesch, G., Anderson, A. & Svec, H. A secondary isotopic standard for 6Li/7Li determinations. *International Journal of Mass Spectrometry and Ion Physics* **12**, 265-272 (1973).
14. Carignan, J., Vigier, N. & Millot, R. Three Secondary Reference Materials for Lithium Isotope Measurements: Li7-N, Li6-N and LiCl-N Solutions. *Geostandards and Geoanalytical Research* **31**, 7-12 (2007).
15. Imai, N., Terashima, S., Itoh, S. & Ando, A. 1994 compilation of analytical data for minor and trace elements in seventeen GSJ geochemical reference samples,"igneous rock series". *Geostandards Newsletter* **19**, 135-213 (1995).
16. Vigier, N. *et al.* Quantifying Li isotope fractionation during smectite formation and implications for the Li cycle. *Geochimica et Cosmochimica Acta* **72**, 780-792 (2008).
17. Luo, X., Rehkämper, M., Lee, D.-C. & Halliday, A. N. High precision 230 Th/232 Th and 234 U/238 U measurements using energyfiltered ICP magnetic sector multiple collector mass spectrometry. *International Journal of Mass Spectrometry and Ion Processes* **171**, 105-117 (1997).
18. Richter, S. *et al.* Improvements in routine uranium isotope ratio measurements using the modified total evaporation method for multi-collector thermal ionization mass spectrometry. *Journal of Analytical Atomic Spectrometry* **26**, 550-564 (2011).
19. Flanagan, F. J. *Descriptions and analyses of eight new USGS rock standards*. (US Government Printing Office, 1976).

20 Sims, K. W. *et al.* An inter-laboratory assessment of the thorium isotopic composition of synthetic and rock reference materials. *Geostandards and Geoanalytical Research* **32**, 65-91 (2008).

21 Verney-Carron, A., Vigier, N. & Millot, R. Experimental determination of the role of diffusion on Li isotope fractionation during basaltic glass weathering. *Geochimica et Cosmochimica Acta* **75**, 3452-3468 (2011).

22 Dellinger, M., Bouchez, J., Gaillardet, J., Faure, L. & Moureau, J. Tracing weathering regimes using the lithium isotope composition of detrital sediments. *Geology* **45**, 411-414 (2017).

23 Millot, R., Vigier, N. & Gaillardet, J. Behaviour of lithium and its isotopes during weathering in the Mackenzie Basin, Canada. *Geochimica et Cosmochimica Acta* **74**, 3897-3912 (2010).

24 Liu, X.-M., Wanner, C., Rudnick, R. L. & McDonough, W. F. Processes controlling δ7Li in rivers illuminated by study of streams and groundwaters draining basalts. *Earth and Planetary Science Letters* **409**, 212-224 (2015).

25 Dosseto, A. *et al.* Rapid response of silicate weathering rates to climate change in the Himalaya. *Geochemical Perspectives Letters* **1**, 10-19 (2015).

26 Decarreau, A. *et al.* Partitioning of lithium between smectite and solution: An experimental approach. *Geochimica et Cosmochimica Acta* **85**, 314-325 (2012).

27 Tessier, A., Campbell, P. G. & Bisson, M. Sequential extraction procedure for the speciation of particulate trace metals. *Analytical chemistry* **51**, 844-851 (1979).

28 Plater, A., Ivanovich, M. & Dugdale, R. Uranium series disequilibrium in river sediments and waters: the significance of anomalous activity ratios. *Applied Geochemistry* **7**, 101-110 (1992).

29 Burton, K. W. & Vigier, N. in *Handbook of Environmental Isotope Geochemistry: Vol I* (ed Mark Baskaran) 41-59 (Springer Berlin Heidelberg, 2012).

30 Williams, L. B. & Hervig, R. L. Lithium and boron isotopes in illite-smectite: the importance of crystal size. *Geochimica et Cosmochimica Acta* **69**, 5705-5716 (2005).

31 Singer, A. & Stoffers, P. Clay mineral diagenesis in two East African lake sediments. *Clay Minerals* **15**, 291-307 (1980).

32 Andronopoulos, V. Herson Sheet (IGME, 1990).

33 Ivanovski, T. List Gevgelija (Savezni geološki institut, 1970).

34 Ma, L. *et al.* Regolith production rates calculated with uranium-series isotopes at Susquehanna/Shale Hills Critical Zone Observatory. *Earth and Planetary Science Letters* **297**, 211-225 (2010).

35 Dosseto, A., Turner, S. P. & Chappell, J. The evolution of weathering profiles through time: new insights from uranium-series isotopes. *Earth and Planetary Science Letters* **274**, 359-371 (2008).

36 Suresh, P., Dosseto, A., Hesse, P. & Handley, H. Soil formation rates determined from Uranium-series isotope disequilibria in soil profiles from the southeastern Australian highlands. *Earth and Planetary Science Letters* **379**, 26-37 (2013).

37 Gontier, A. *et al.* Lack of bedrock grain size influence on the soil production rate. *Geochimica et Cosmochimica Acta* **166**, 146-164 (2015).

38 Thienemann, M. *et al.* Organic geochemical and palynological evidence for Holocene natural and anthropogenic environmental change at Lake Dojran (Macedonia/Greece). *The Holocene* **27(8)**, 1103-1114 (2017).

Table S1: Radiocarbon and calendar ages from core Co1260. The calibration of radiocarbon ages into calendar ages is based the IntCal13 calibration curve[5] and on a 2σ uncertainty level.

AMS Lab ID	core depth [cm]	material	C weight [mg]	^{14}C age [yr BP]	calendar age range [cal yr BP]
COL 1312.1.1	16.5	bulk organic C	0.44	140 ± 35	5 to 280
COL 1324.1.1	53.3	terrestrial plant	0.45	360 ± 70	300 to 515
COL 1314.1.1	111.3	terrestrial plant	0.32	840 ± 70	675 to 910
COL 1194.1.1	253.0	terrestrial plant	1.00	2430 ± 30	2355 to 2685
COL 1316.1.1	287.3	terrestrial plant	1.00	3080 ± 30	3715 to 3365
COL 1317.1.1	309.1	charcoal	1.00	3560 ± 40	3725 to 3970
ETH 44956.1.1	404.9	carbonate	1.01	6410 ± 40	7280 to 7420
COL 1319.1.1	406.4	terrestrial plant	0.28	8020 ± 150	8540 to 9370
COL 1320.1.1	460.9	terrestrial plant	0.63	9520 ± 160	10410 to 1225
ETH 44957.1.1	502.9	carbonate	1.00	9840 ± 40	11200 to 11310
COL 1321.1.1	521.9	terrestrial plant	0.90	9330 ± 160	10230 to 11090
ETH 46615.1.1	635.0	bulk organic C	1.00	10 220 ± 70	11620 to 12370
ETH 44958.1.1	663.9	carbonate	0.48	28 570 ± 170	31900 to 33280

Table S2: Interpolated ages as well as their associated error bars of the samples selected for isotope analyses. The errors were obtained at 95% confidence level using the software package clam2.2[(4)].

Depth [cm]	Age [cal yr BP]		
	min95%	max95%	best
1	-67	-25	-48
73.3	411	645	532
131.3	778	1093	915
185.3	1203	1695	1400
251.3	2329	2645	2438
261.3	2568	2817	2658
269.3	2758	2971	2845
278.9	2993	3160	3077
298.9	3515	3646	3576
308.9	3768	3964	3865
316.9	4021	4256	4141
330.9	4573	4861	4715
346.9	5310	5664	5481
358.9	5920	6301	6110
374.9	6808	7152	6988
386.9	7542	7757	7654
396.9	8183	8225	8200
410.9	8721	9109	8901
422.9	9080	9729	9379
432.9	9329	10133	9694
456.9	9762	10740	10200
480.9	10025	11015	10453
514.9	10246	11127	10624
546.9	10496	11298	10844
602.9	11260	11739	11492
682.1	12057	13105	12605

Table S3: Lithium isotope measurements of reference materials.

Sample	δ^7Li vs L-SVEC [‰]	Reference
Li7-N (synthetic)	30.4 ± 0.1 (n=29)	this study
Li7-N	30.2 ± 0.3 (n=89)	[14]
Li7-N	30.2 ± 0.3 (n=13)	[16]
Li6-N (synthetic)	-7.8 ± 0.1 (n=31)	this study
Li6-N	-8.0 ± 0.3 (n=38)	[14]
Li6-N	-8.9 ± 0.9 (n=9)	[16]
JG-2 (granite)	0.6 ± 0.1 (n=22)	this study
JG-2 (granite)	0.4 ± 0.2 (n=3)	[11]
JG-2 (granite)	0.2 ± 0.1	[16]

Uncertainties are given at the 2σ level.

Table S4: Lithium and uranium isotope compositions in lake sediments.

Sample name	Depth [cm]	Age [cal yr BP]	$\delta^{7}Li$ [‰]	$\delta^{234}U$ [‰]
Dojran 1	1	0	-0.2	0
Dojran 65	73.3	500	0.1	-18
Dojran 94	131.3	1030	0.6	-12
Dojran 121	185.3	1610	-0.1	-5
Dojran 154	251.3	2550	0.3	-3
Dojran 159	261.3	2720	-0.6	-6
Dojran 163	269.3	2840	-1.0	-18
Dojran 189	278.9	3070	-1.9	-33
Dojran 199	298.9	3530	-2.8	-42
Dojran 204	308.9	3810	-2.4	-31
Dojran 208	316.9	4060	-2.6	-25
Dojran 215	330.9	4570	-2.0	-15
Dojran 223	346.9	5330	-1.7	-2
Dojran 229	358.9	6060	-1.9	-16
Dojran 229 replicate			-1.4	
Dojran 237	374.9	7070	-1.6	-24
Dojran 243	386.9	7690	-1.1	-18
Dojran 248	396.9	8110	-1.6	-39
Dojran 248 replicate			-1.6	
Dojran 255	410.9	8590	-0.6	-2
Dojran 261	422.9	8940	-0.8	4
Dojran 266	432.9	9190	-1.1	-16
Dojran 278	456.9	9700	-0.5	-11
Dojran 290	480.9	10120	-0.7	-22
Dojran 307	514.9	10620	0.4	-26
Dojran 348	546.9	11020	0.6	-26
Dojran 376	602.9	11610	0.3	-19
Dojran 460	682.1	12280	-0.2	-34

External uncertainty is 1.8 and 0.4 ‰ for $\delta^{234}U$ and $\delta^{7}Li$, respectively.

Table S5: Lithium isotope compositions of stream sediments.

Sample name	δ^7Li [‰]
Dojran stream #1	-0.4
Dojran stream #7	-0.7
Dojran stream #8	-1.4
Dojran stream #9	-4.1
Dojran stream #12	-4.8
Dojran stream #15	-4.4
Dojran stream #18	-0.0
Dojran stream #19	-0.3
Dojran stream #22	0.8
Dojran stream #22 replicate	0.5
Dojran stream #35	-0.4

External uncertainty of δ^7Li values is 0.4 ‰.

Table S6: Grain size data and sediment K/Ti ratios of lake sediments. Sample depth is the mean over 2 cm thick sediment layers.

**Sample name**	**Depth [cm]**	**Age [cal yr BP]**	**Mean grain size [µm]**	**K/Ti**
Dojran 1	1	0	24	5.2
Dojran 65	73.3	500	18	5.2
Dojran 94	131.3	1030	18	4.9
Dojran 121	185.3	1610	16	5.6
Dojran 154	251.3	2550	16	4.9
Dojran 159	261.3	2720	15	4.8
Dojran 163	267.3	2840	18	4.7
Dojran 189	278.9	3070	19	4.4
Dojran 199	298.9	3530	19	5.6
Dojran 204	308.9	3810	18	4.4
Dojran 208	316.9	4060	22	3.9
Dojran 215	330.9	4570	23	3.8
Dojran 223	346.9	5330	22	3.7
Dojran 229	358.9	6060	24	3.9
Dojran 237	374.9	7070	20	4.0
Dojran 243	386.9	7690	21	4.2
Dojran 248	396.9	8110	20	4.6
Dojran 255	410.9	8590	19	4.3
Dojran 261	422.9	8940	21	4.1
Dojran 266	432.9	9190	17	4.6
Dojran 278	456.9	9700	19	4.7
Dojran 290	480.9	10120	21	4.6
Dojran 307	514.9	10620	22	5.1
Dojran 348	546.9	11020	19	5.6
Dojran 376	602.9	11610	17	6.0
Dojran 460	682.1	12280	23	4.4

Deposition ages were determined using radiocarbon. Uncertainty levels are in detail discussed in Francke et al. 2013[2]. External uncertainty for K/Ti ratios is 0.6, calculated from two replicate samples. Error on mean grain size data has not been determined.

Table S7: Mineralogy of lake sediments.

Sample name	Depth [cm]	Age [cal yr BP]	Augite [%]	Biotite [%]	Calcite [%]	Chlorite [%]	Dolomite [%]	Epidote [%]	Forsterite [%]	Muscovite [%]	Orthoclase [%]	Quartz [%]	Talc [%]
Dojran 1	1	0	3.7	13.6	5.4	7.9	0.3	0.3	0	40.3	11.3	13.2	3.9
Dojran 65	73.3	500	10.1	17.9	5.6	9	2.7	2.4	1.7	28.1	8.4	14.1	0.1
Dojran 94	131.3	1030	22.6	11.1	11.9	10.5	0.7	5.7	10.4	9.5	2.4	9.4	5.7
Dojran 121	185.3	1610	14.5	13.8	7.4	11.1	2.9	4.1	0	23.3	8.3	11.2	3.4
Dojran 154	251.3	2550	12	15.6	2.4	10.5	2.3	2.9	0	31.2	7.6	15.3	0.1
Dojran 159	261.3	2720	4.8	13.5	3	11.9	0.8	1	0	42.3	6.3	16.3	0
Dojran 163	267.3	2840	3.9	11.8	6	8.5	0.6	1.9	0	39.4	6.3	21.5	0
Dojran 189	278.9	3070	1.1	9.3	22.7	9.2	1.7	5	3.1	23.8	11.6	11.3	1.2
Dojran 199	298.9	3530	8.7	5.5	33.1	7.5	2.5	7.9	3	11.4	5	11.5	3.8
Dojran 204	308.9	3810	0	9.4	30.9	7.3	0	0	0	32.9	5.1	14.2	0
Dojran 208	316.9	4060	10.9	7	37.5	9.9	2.2	4.8	2.6	11.5	1.9	6.3	5.4
Dojran 215	330.9	4570	13.1	8.1	43.6	7.1	0.7	2.2	2.7	11.4	3.7	7.2	0.3
Dojran 223	346.9	5330	1.6	5	54.8	4.9	0.4	2.7	0	13.4	7	9.8	0.4
Dojran 229	358.9	6060	5.6	9.8	30.3	6.1	2	8.6	1.9	28.1	5.5	2.1	0.1
Dojran 237	374.9	7070	5.2	10.1	34.1	9.9	3.3	9.7	5.1	15.6	2.3	4.6	0.1
Dojran 243	386.9	7690	2.3	8.6	31.4	5.8	0	0	0	30.4	9.1	12.5	0
Dojran 248	396.9	8110	20.8	13.2	16.7	10.5	0.6	6.8	5.5	9	2.4	6.9	7.6
Dojran 255	410.9	8590	1.8	7.7	50.8	5.2	0.3	1.4	0.7	19	4.6	8.4	0.2
Dojran 261	422.9	8940	1.8	4.8	48.7	3.7	0.5	1.9	0	15.2	2.2	21.1	0
Dojran 266	432.9	9190	0.3	7.3	45.6	6.3	0.9	3.5	1.8	16.6	9.3	5.4	3
Dojran 278	456.9	9700	1.2	10.6	35.5	5.8	0.6	0	0	25.3	6.6	14.4	0
Dojran 290	480.9	10120	0	10.3	37.1	4.5	1.6	3.1	0	23.8	5.4	11.2	3
Dojran 307	514.9	10620	0	15.9	22.4	6.7	0.2	0	0	38.6	6	10.3	0
Dojran 348	546.9	11020	12.5	12.9	19.7	10	1.3	1.8	2.4	19.9	7.1	12.3	0.1
Dojran 376	602.9	11610	8.9	8.6	20.2	6.6	0.8	7.4	2.7	15.1	15.3	10.5	3.9
Dojran 460	682.1	12280	6.7	14	14	13.7	2	6.6	0	23.2	4.4	14.2	1

Relative errors on mineral contents are less than 5% (2σ).

Table S8: Lithium isotope compositions of hand-picked biotite grains

Sample name	δ^7Li [‰]
Dojran stream #22 Biotite (A)	2.4
Dojran stream #22 Biotite (B)	2.0

External uncertainty for δ^7Li values is 0.4‰.

Table S9: Lithium isotope compositions of leached and un-leached samples

Sample name	δ^7Li [‰]
Clay_1	-1.0
Clay_1_leached	-0.4
Clay_2	-0.4
Clay_2_leached	-0.1

Leached clay samples were treated with 8mL 1M NH_4Cl for one hour at room temperature. External uncertainty for δ^7Li values is 0.4‰.

Table S10: Uranium isotope compositions of bedrock samples (see map Fig. S16)

Sample name	$\delta^{234}U$ [‰]	Rock type
Lithology 1	-167	Gneiss
Lithology 2	-13	Phyllite
Lithology 3	-25	Greenschist
Lithology 4	55	Marble
Lithology 5	-58	Phyllite
Lithology 6	67	Volcanoclastic sedimentary rock
Lithology 7	-5	Peridotite
Lithology 8	84	Gneiss
Lithology 9	-40	Granite
Lithology 10	-8	Gneiss
Lithology 11	7	Gabbro

External uncertainty is 1.8 ‰ for $\delta^{234}U$.

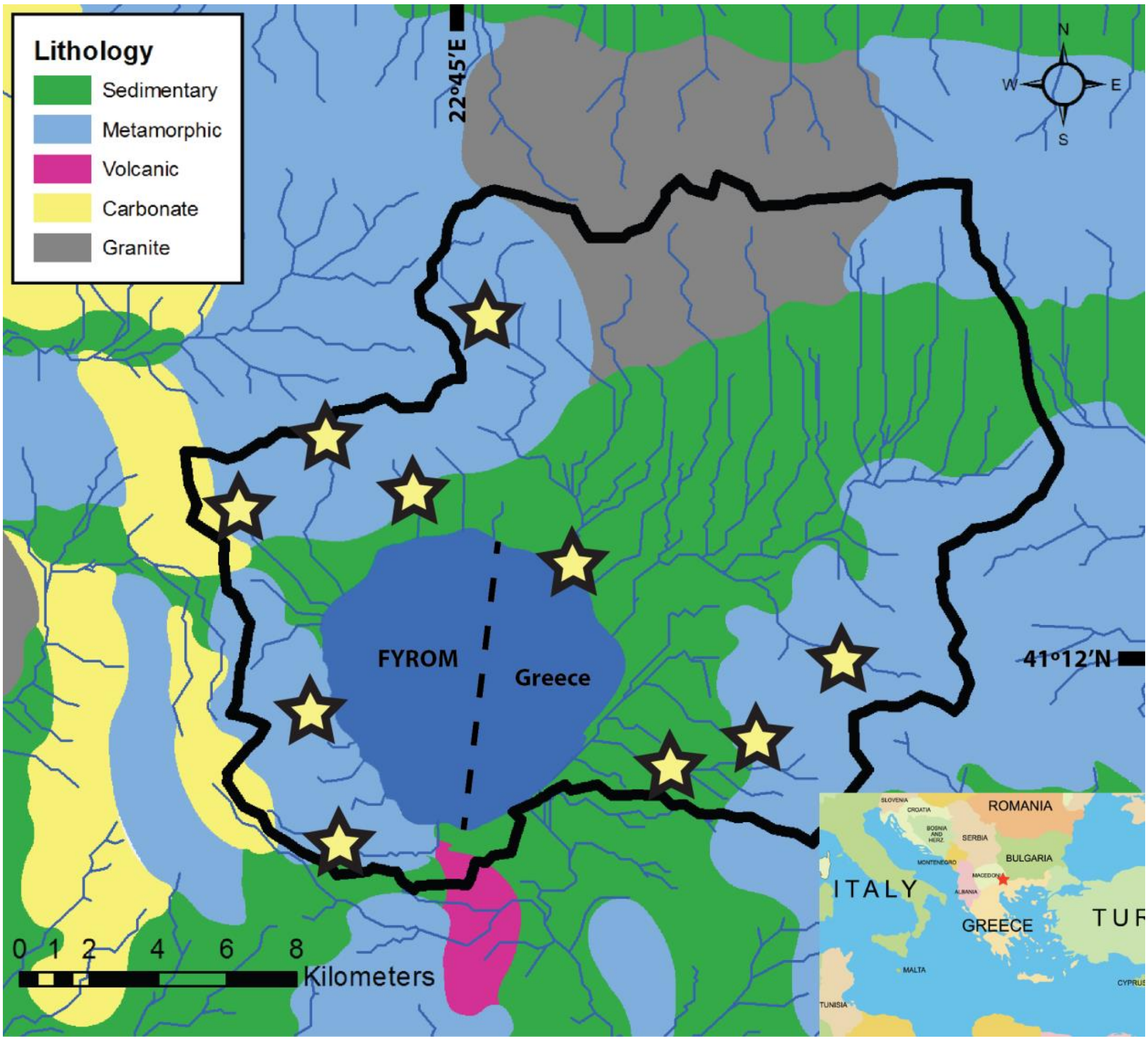

Figure S1: Simplified distribution of geological units for Lake Dojran catchment based on field mapping by A.F. and from ref.[32,33]. Individual lithology areas were estimated and manually drawn by L.R. Black curve: catchment boundary, blue lines: stream network. Dashed line shows the border between FYROM and Greece. Stars show sample locations for stream sediments. Stream network and catchment area was calculated with ArcGIS Desktop Advanced 10.4 software (https://esriaustralia.com.au/arcgis-desktop) using a 1-arc second digital elevation model (DEM). Insert: South-east Europe, star marks location of Lake Dojran (open source: www.vectorworldmap.com Political World Map v. 2.2 (2009)).

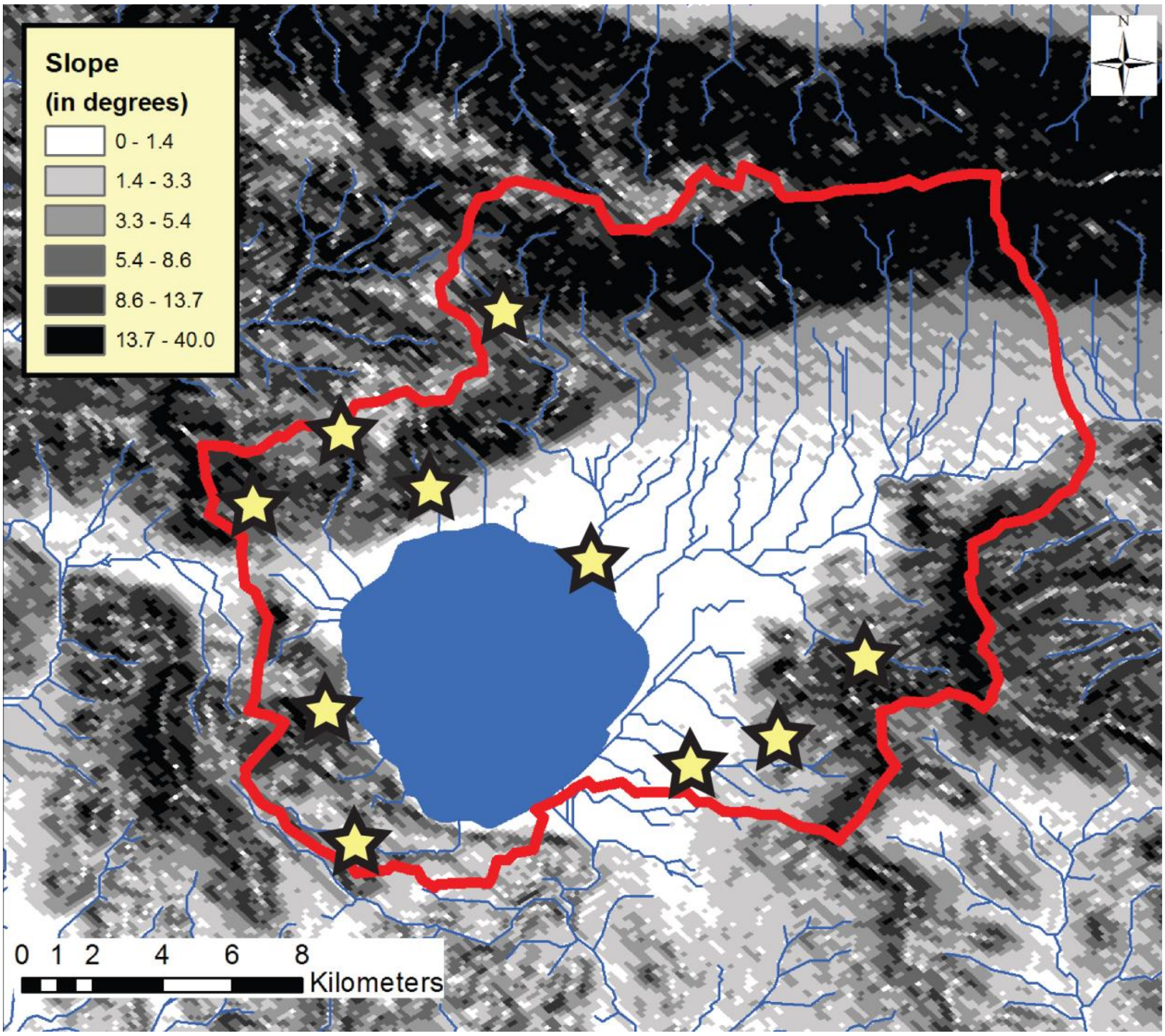

Figure S2: Map of slope for Lake Dojran catchment. Stars show sample locations for stream sediments. Red line is the catchment boundary. Blue lines are representing the modern stream system. Slope model and stream network was calculated with ArcGIS Desktop Advanced 10.4 software (https://esriaustralia.com.au/arcgis-desktop) using a 1-arc second digital elevation model (DEM).

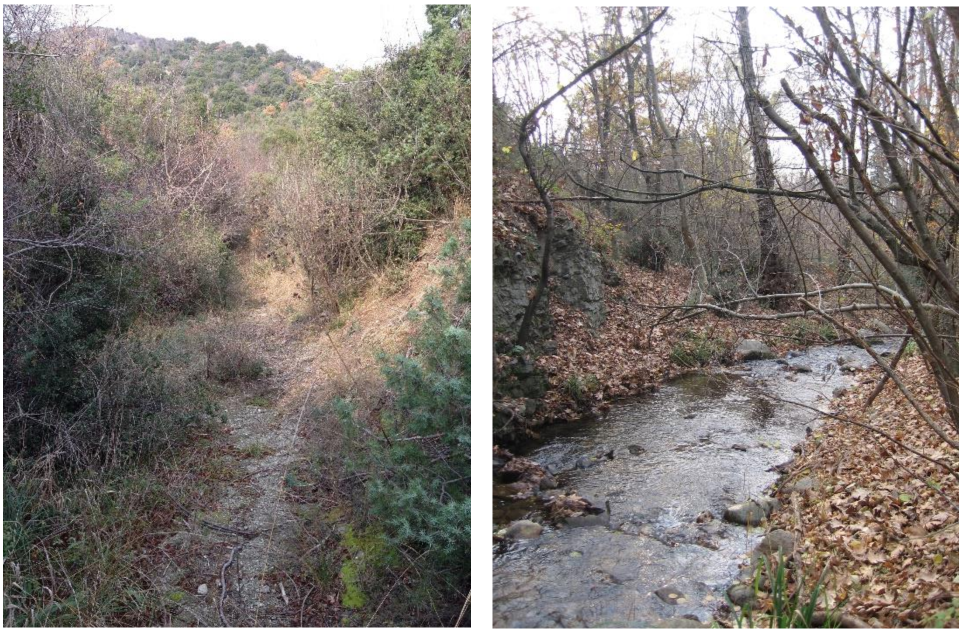

Figure S3: Examples of typical streams flowing into Lake Dojran during winter 2015. Left: stream sample #4. Right: stream sample #1. Channel width on right picture was approximately 2m. Photos were taken by A.F.

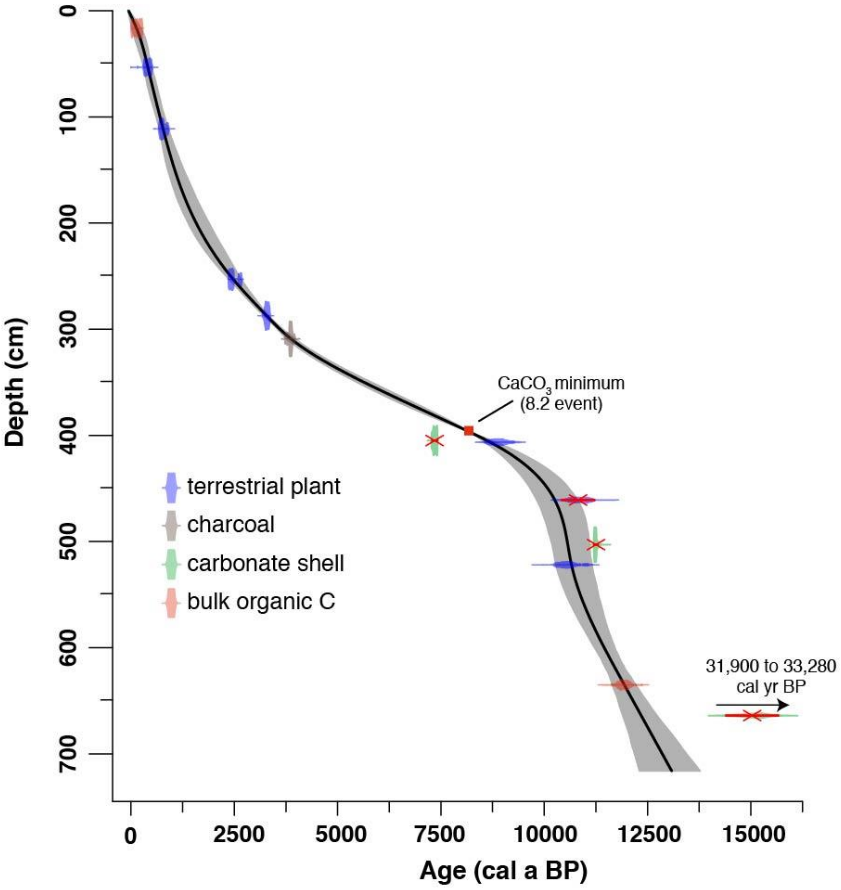

Figure S4: Age-depth model of the analyzed core sequence from Lake Dojran. The age model is based on 13 calibrated radiocarbon ages derived from terrestrial plant material, charcoal, carbonates shell fragments, and bulk organic C samples. Additionally, the 8.2 ka cooling event described from nearby lakes Ohrid and Prespa was correlated with the minimum in $CaCO_3$ at 397 cm depth. Samples marked with a red cross were excluded from the age depth interpolation as they are

considered to be affected by reservoir and/or hard water effects (carbonate shell sample at 502 cm depth), were relocated during core opening (terrestrial plant material sample at 460 cm depth and carbonate shell sample at 404.9 cm depth), or were re-deposited prior to final deposition (carbonate sample at 663.9 cm depth)

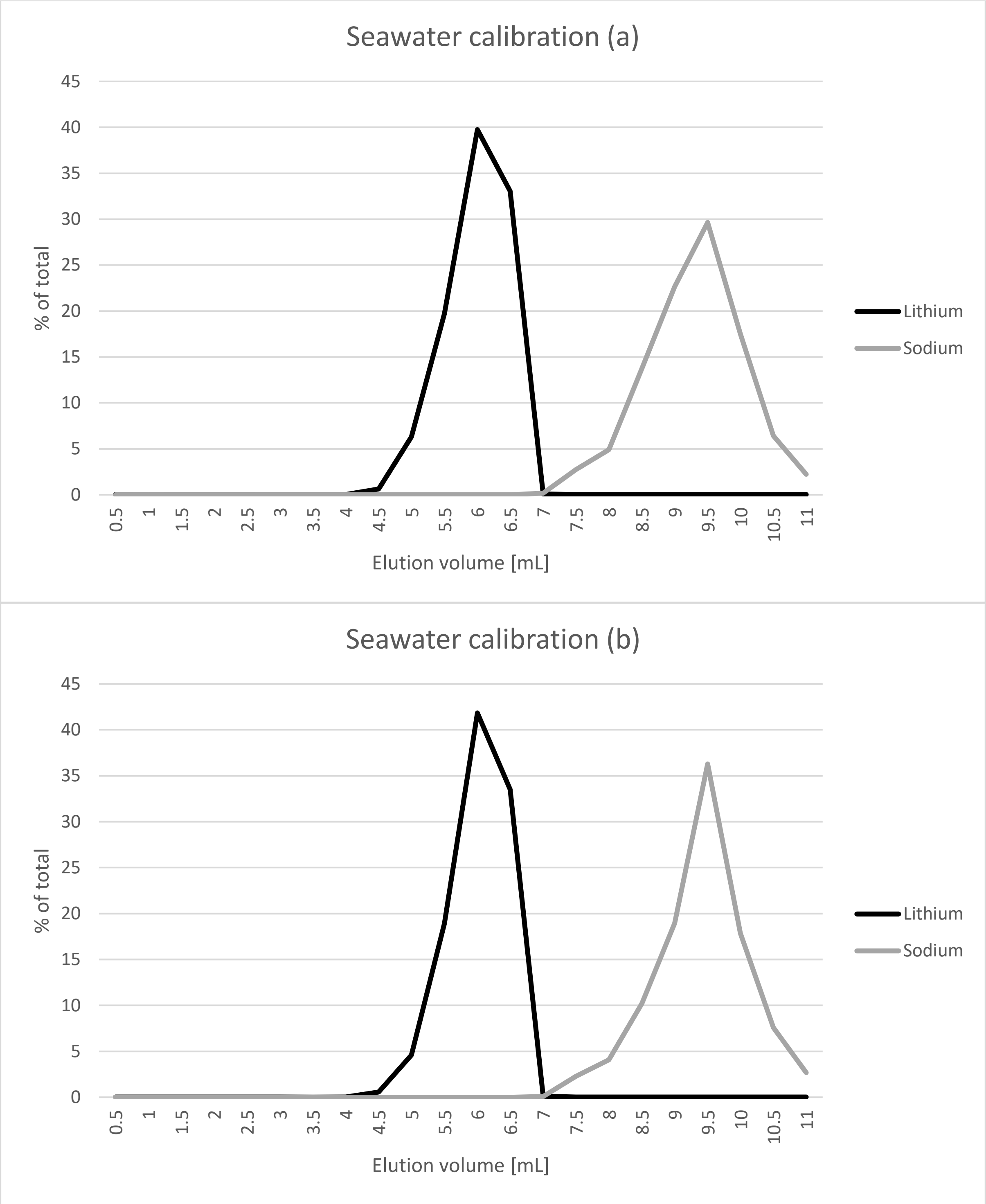

Figure S5: Elution of Li and Na for seawater on two individual columns. Both (a) and (b) show results from first pass through the cation exchange column. Elution reagent is 1M HCl. Lithium was collected from 4.5 – 8 mL.

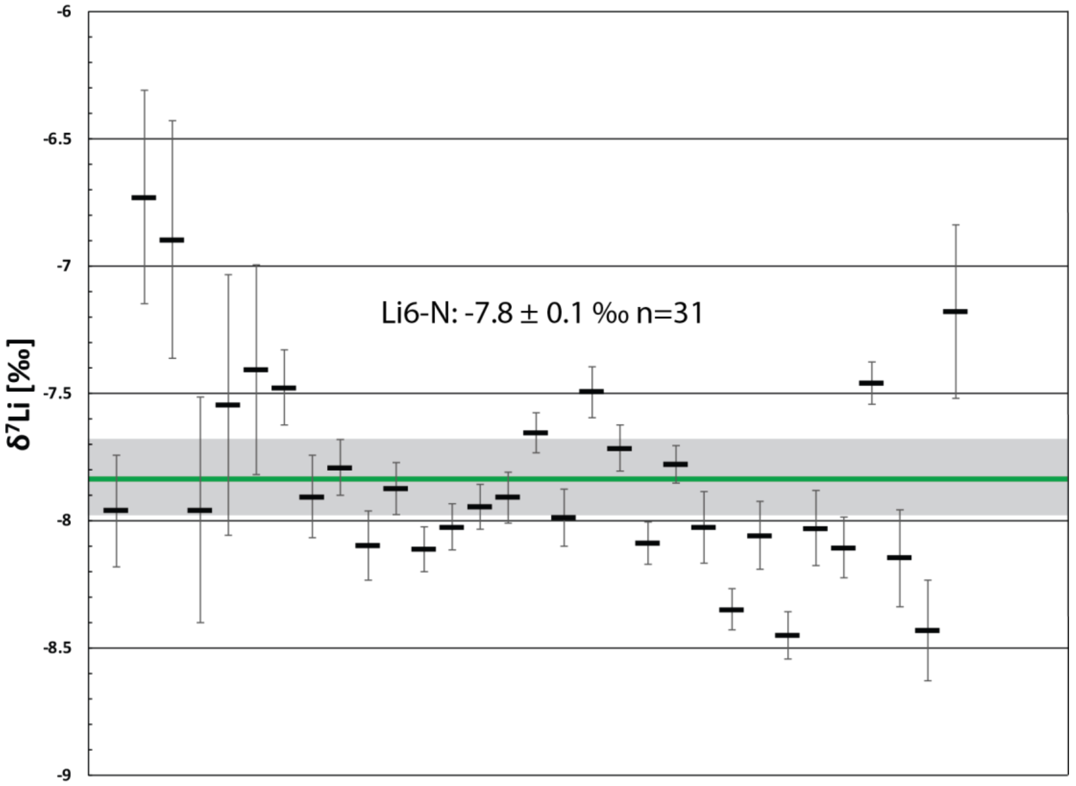

δ7Li [‰]
Li6-N: -7.8 ± 0.1 ‰ n=31
-6
-6.5
-7
-7.5
-8
-8.5
-9

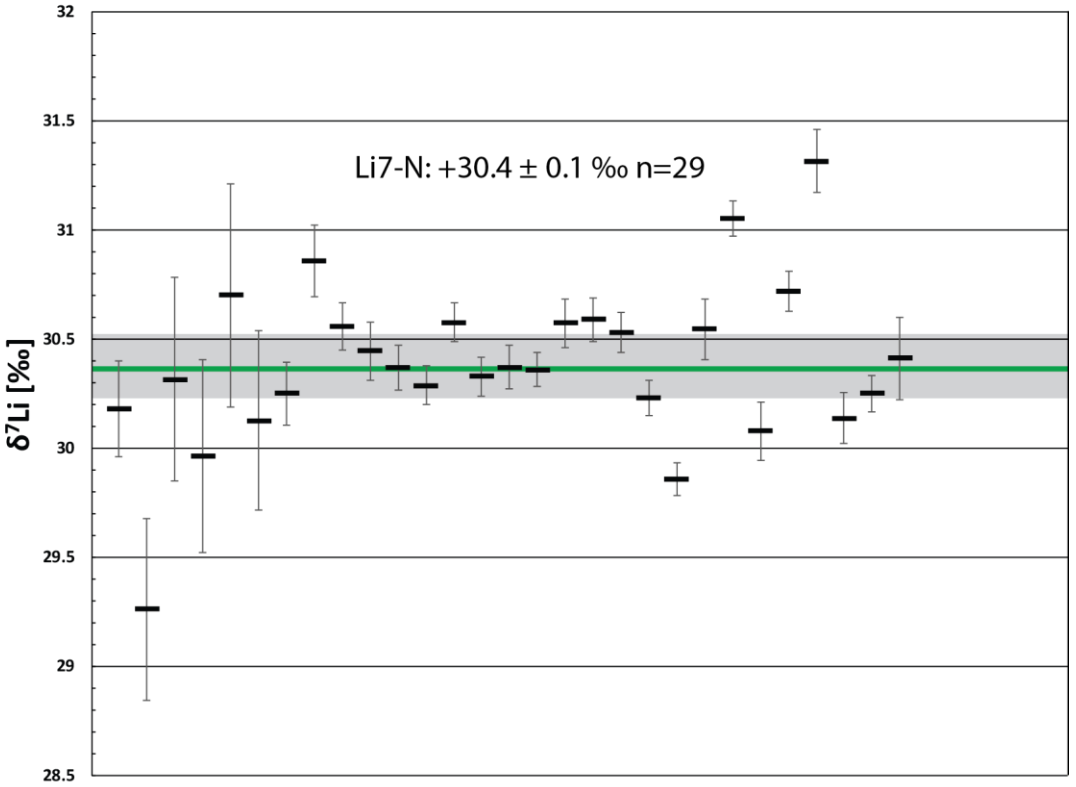

δ7Li [‰]
Li7-N: +30.4 ± 0.1 ‰ n=29
32
31.5
31
30.5
30
29.5
29
28.5

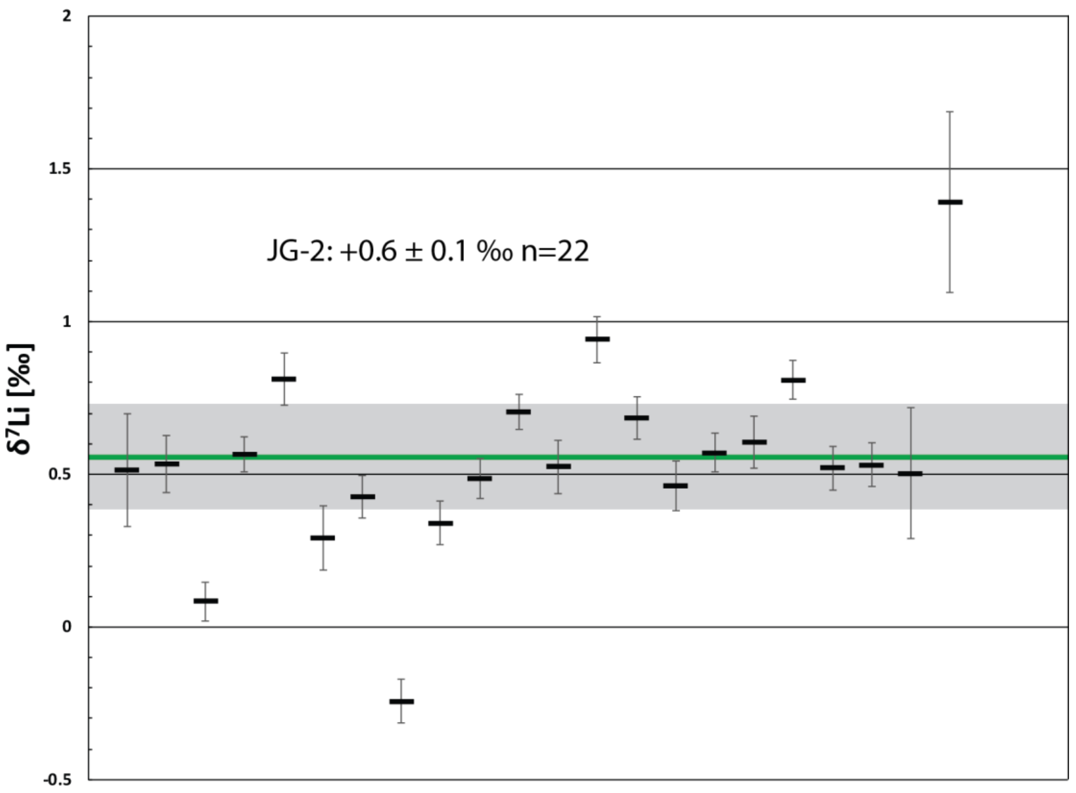

Figure S6: Lithium isotope measurements for synthetic secondary reference material Li6-N, Li7-N[14] and a granite rock standard JG-2[15] using a MC ICP-MS at Wollongong Isotope Geochronology Laboratory at University of Wollongong. Error bars for each measurement are 2 standard error (internal uncertainty). Green line is the average value out of all measurements, of which the grey band is the 2SE.

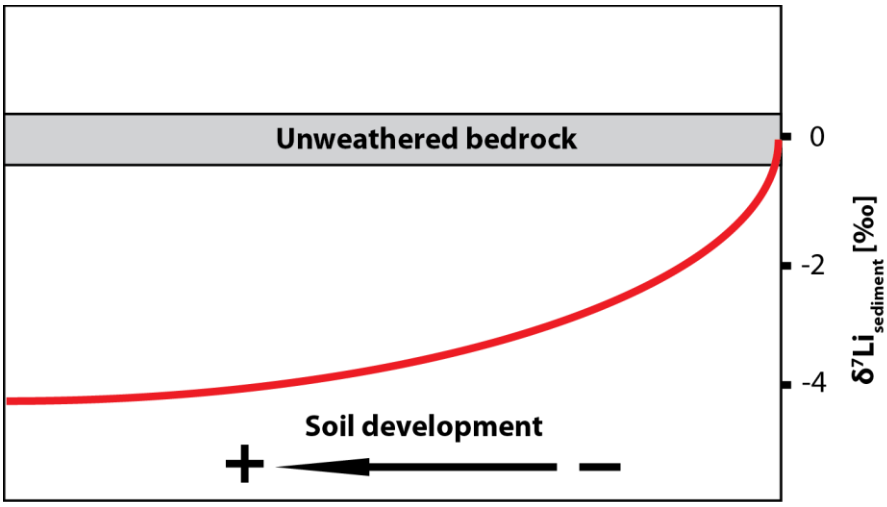

Figure S7: Conceptual representation of the evolution of lithium isotope composition in sediments (red curve) as a result of increasing clay neo-formation and soil development. Sediments mixture of primary and secondary phases, exhibit low δ^7Li where clays are more abundant relative to primary minerals. In contrast, sediments recording more positive δ^7Li are likely to consist dominantly of primary phases, as a result of little clay neo-formation.

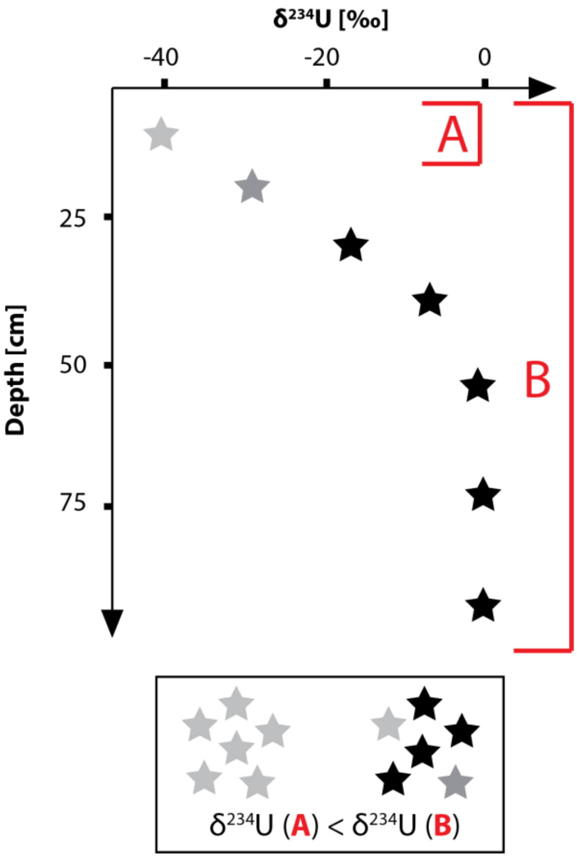

Figure S8: Conceptual representation of uranium isotope compositions in soil profiles. Deep soil horizons have δ^{234}U values close to secular equilibrium i.e. 0‰, whereas upper parts of a soil profile have been exposed to chemical weathering for longer, decreasing δ^{234}U values. If erosion is shallow (e.g. sediment A) and only the top soil is removed, sediments will exhibit a lower δ^{234}U than if erosion is deep and deeper soil material is mobilized (e.g. sediment B).

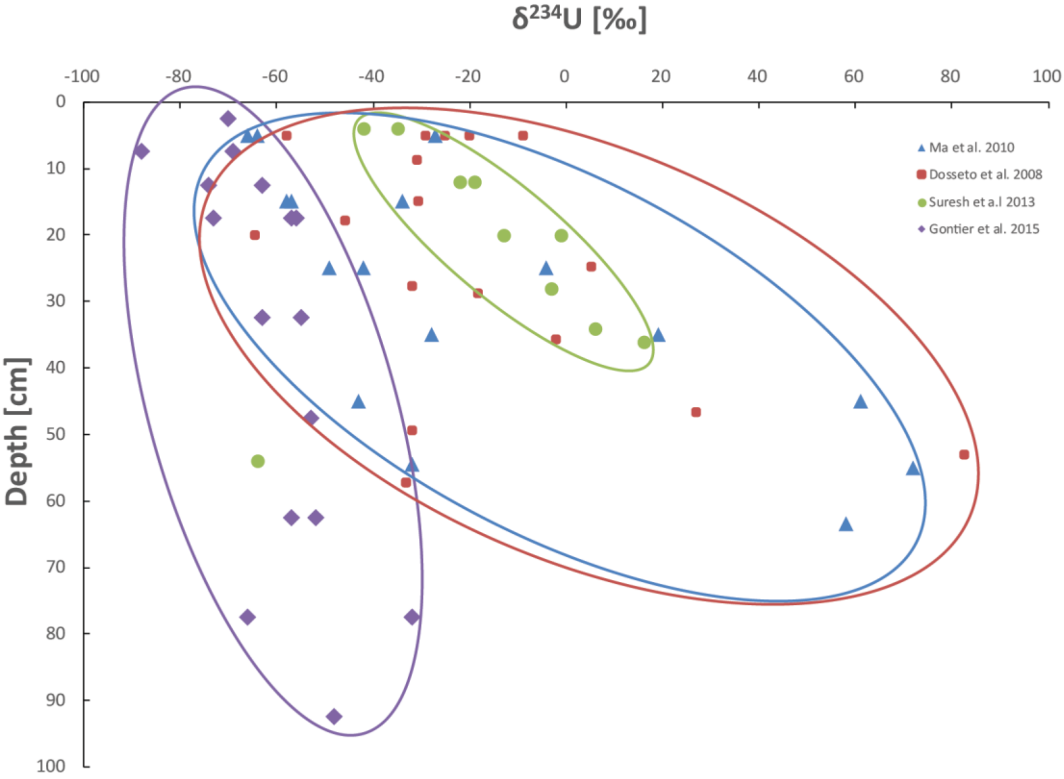

Figure S9: Uranium isotope ratios (in ‰) as a function of depth (cm) in selected soil profiles from the literature[34-37], illustrating how $\delta^{234}U$ decreases with soil depth.

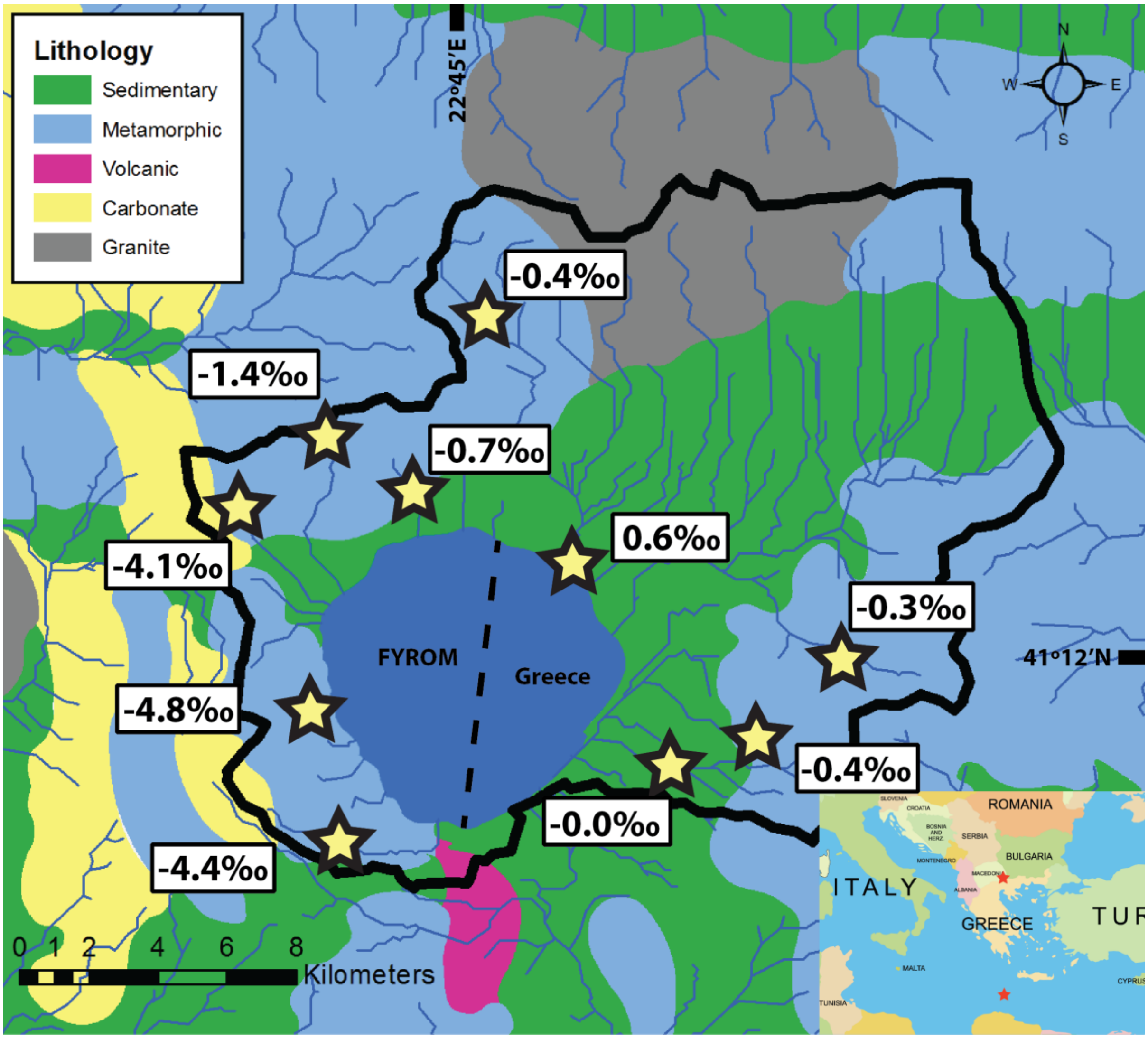

Figure S10: Simplified distribution of geological units for Lake Dojran catchment based on field mapping by A.F. and from ref. [32,33]. Individual lithology areas were estimated and manually drawn by L.R. This map was created with ArcGIS Desktop Advanced 10.4 software (https://esriaustralia.com.au/arcgis-desktop). Stars represent stream sample locations. Displayed values are $\delta^{7}Li$ compositions of stream sediments. External uncertainty of $\delta^{7}Li$ measurements is 0.4 ‰. A replicate was done for sample #22 for which the average $\delta^{7}Li$ value is displayed (0.6 ‰ n=2).

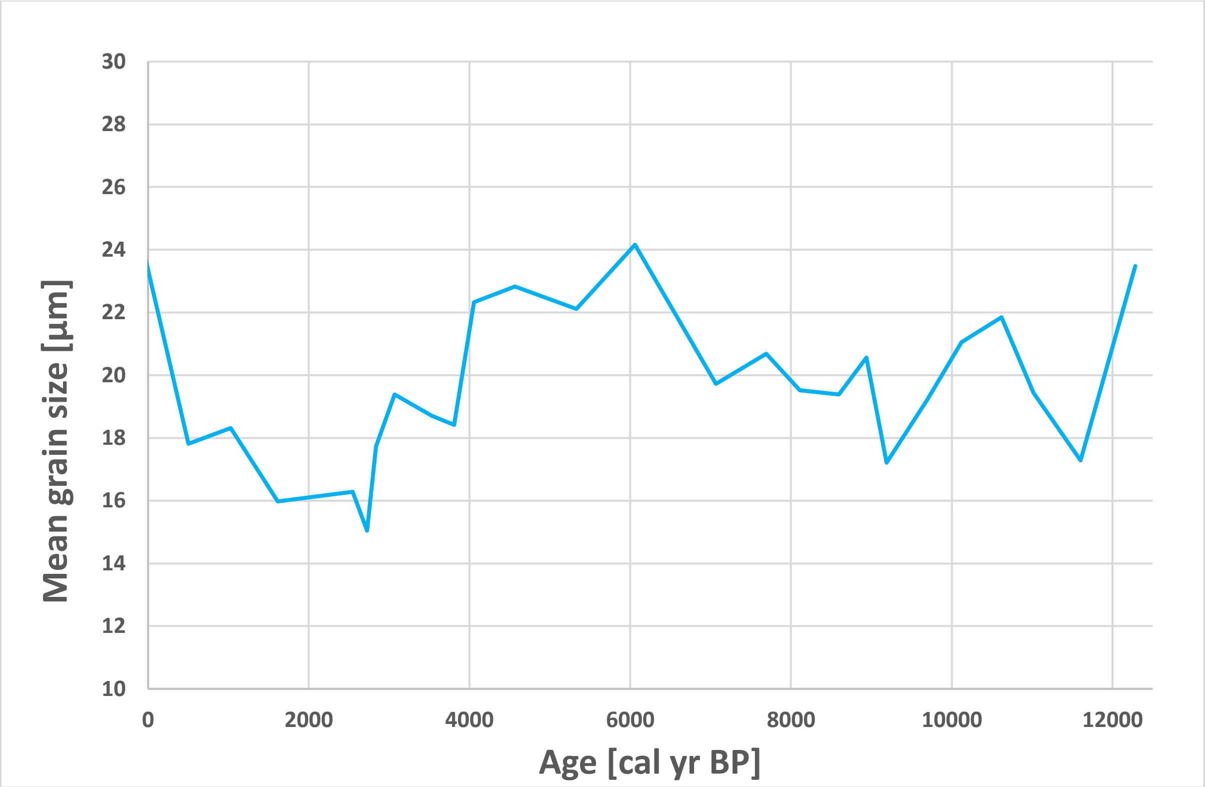

532
533 *Figure S11: Mean grain size of Lake sediments (in µm) as a function of the deposition age (in cal yr BP).*

534

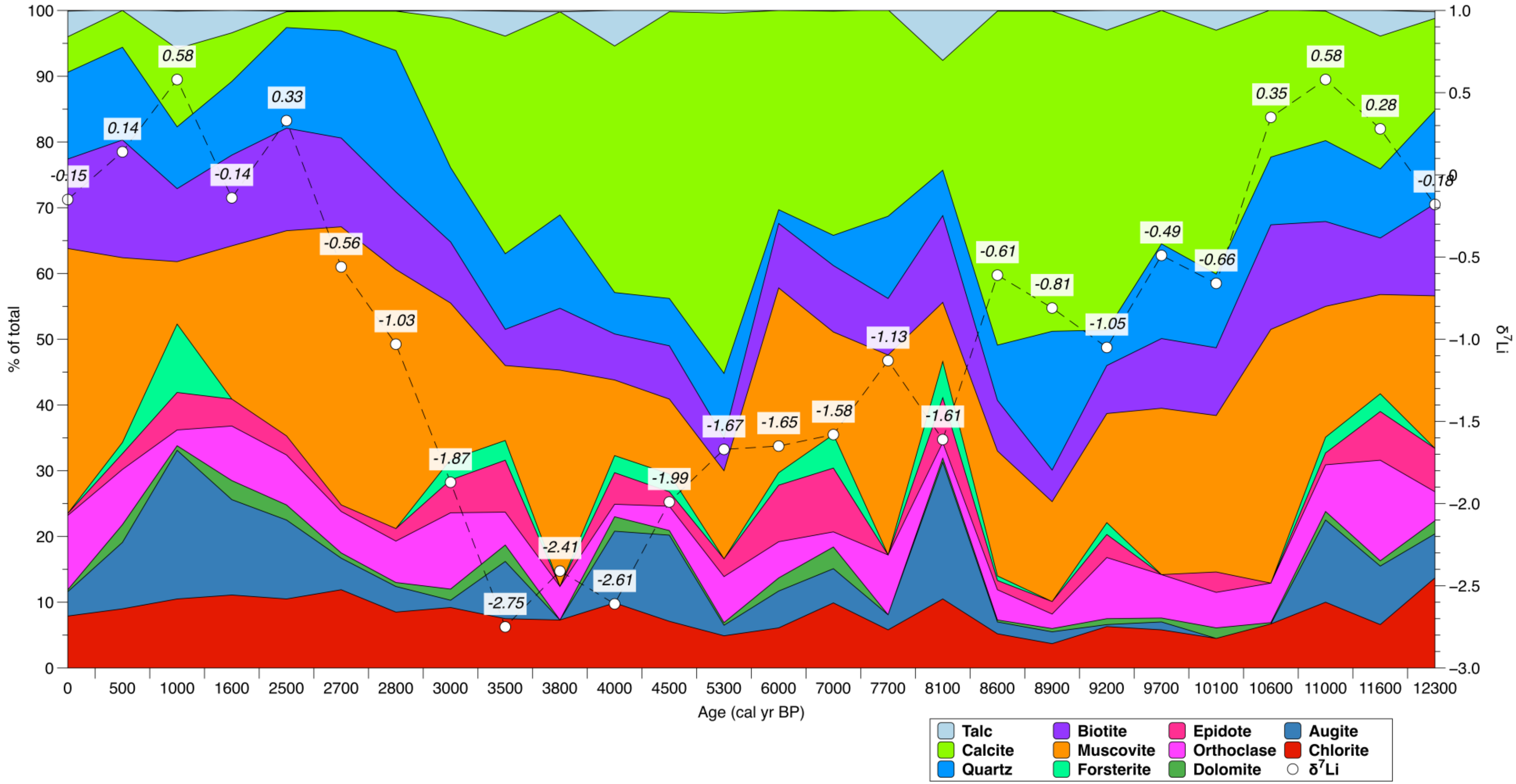

Figure S12: Cumulative mineralogical distribution (in wt %) in lake sediments as a function of depositional ages (in cal yr BP). Open circles: Li isotope compositions (right Y-axis) of Lake sediments with an error of 0.4 ‰ (2SE).

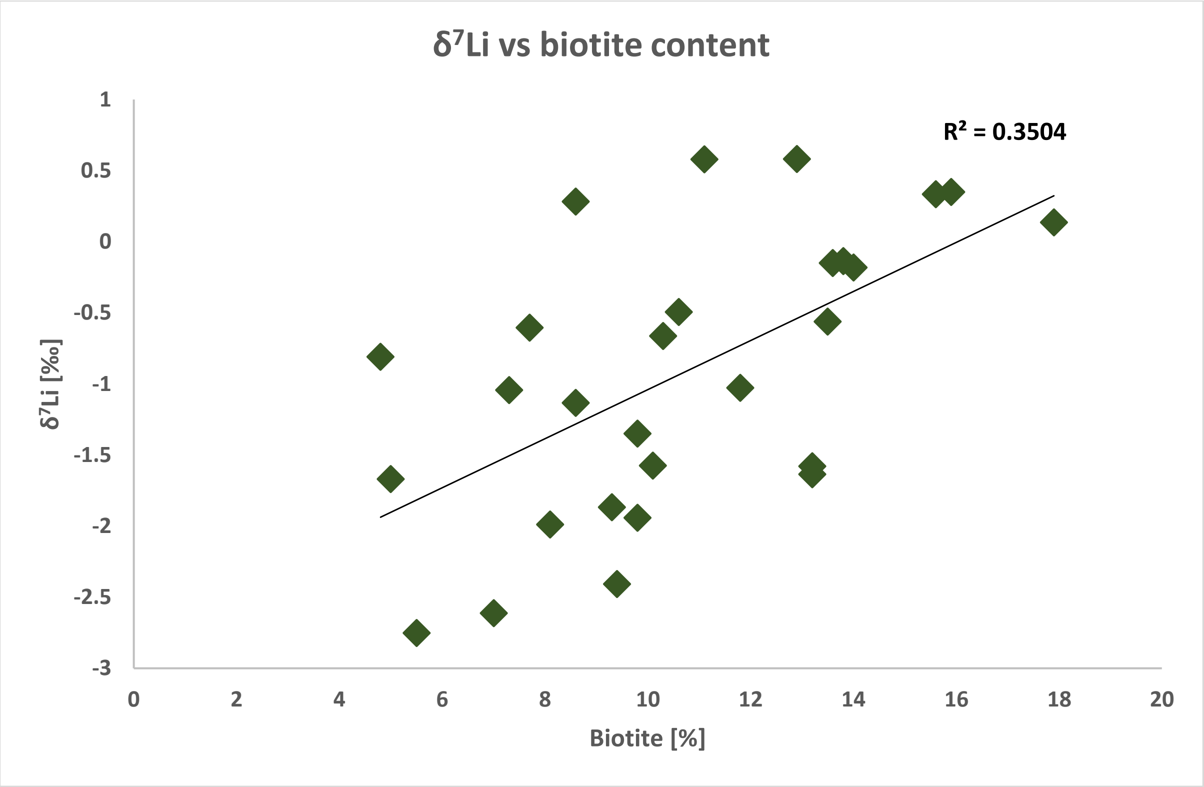

Figure S13: Li isotope ratios in lake sediments (in ‰) as a function of biotite content (in wt %).

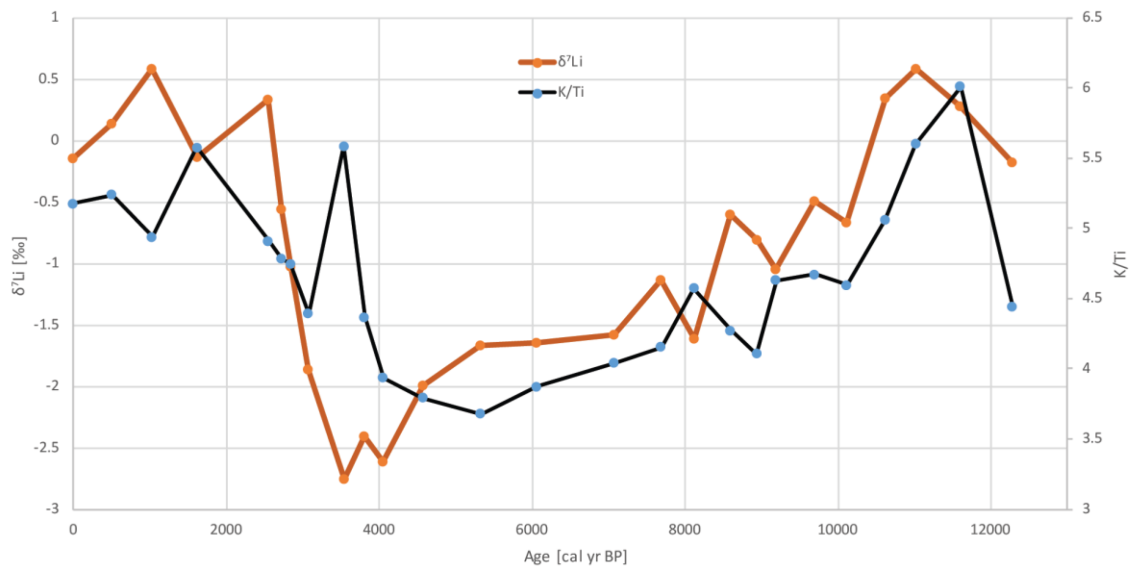

Figure S14: Li isotope compositions (in ‰, red line) and K/Ti ratios (unit less, black line) of lake sediments as a function of deposition age (in cal. yr BP). Both proxies indicate increasing chemical weathering associated with soil development until human impact ~3,000 cal. yr BP.

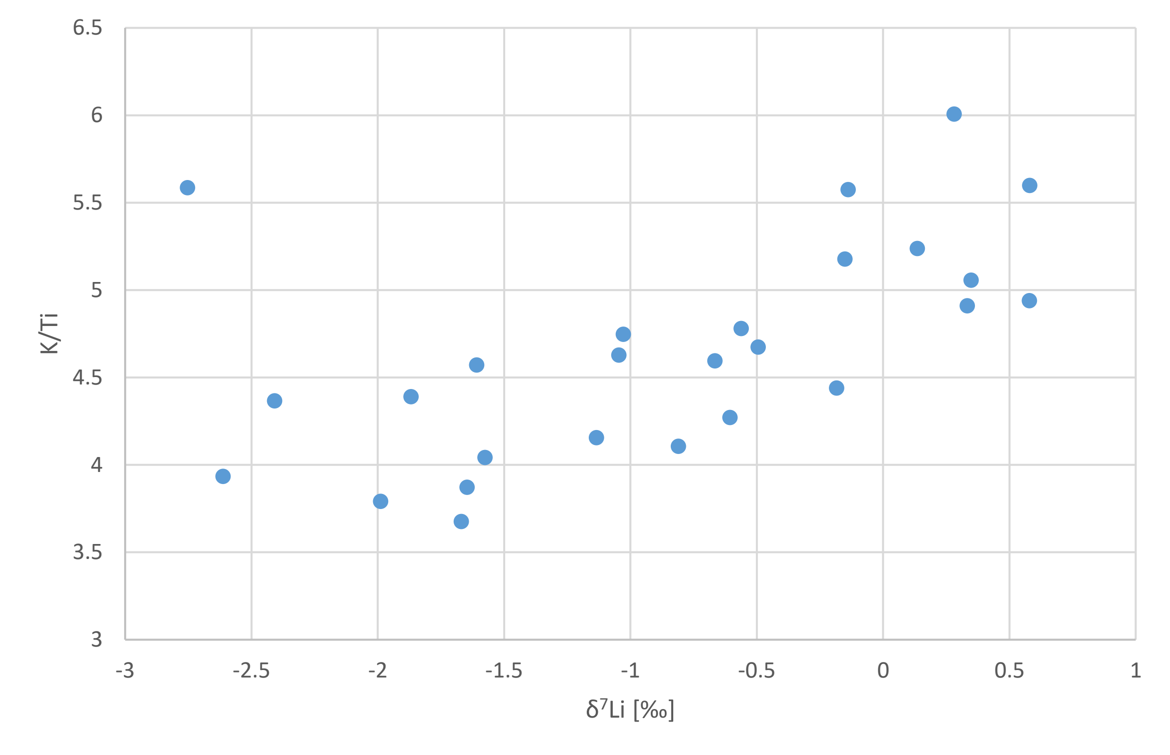

Figure S15: K/Ti ratios (unit less) as a function of δ^7Li compositions (in ‰) of lake sediments. The positive correlation between δ^7Li and K/Ti ratios indicates that lithium isotopes fractionate in concert with chemical weathering reactions.

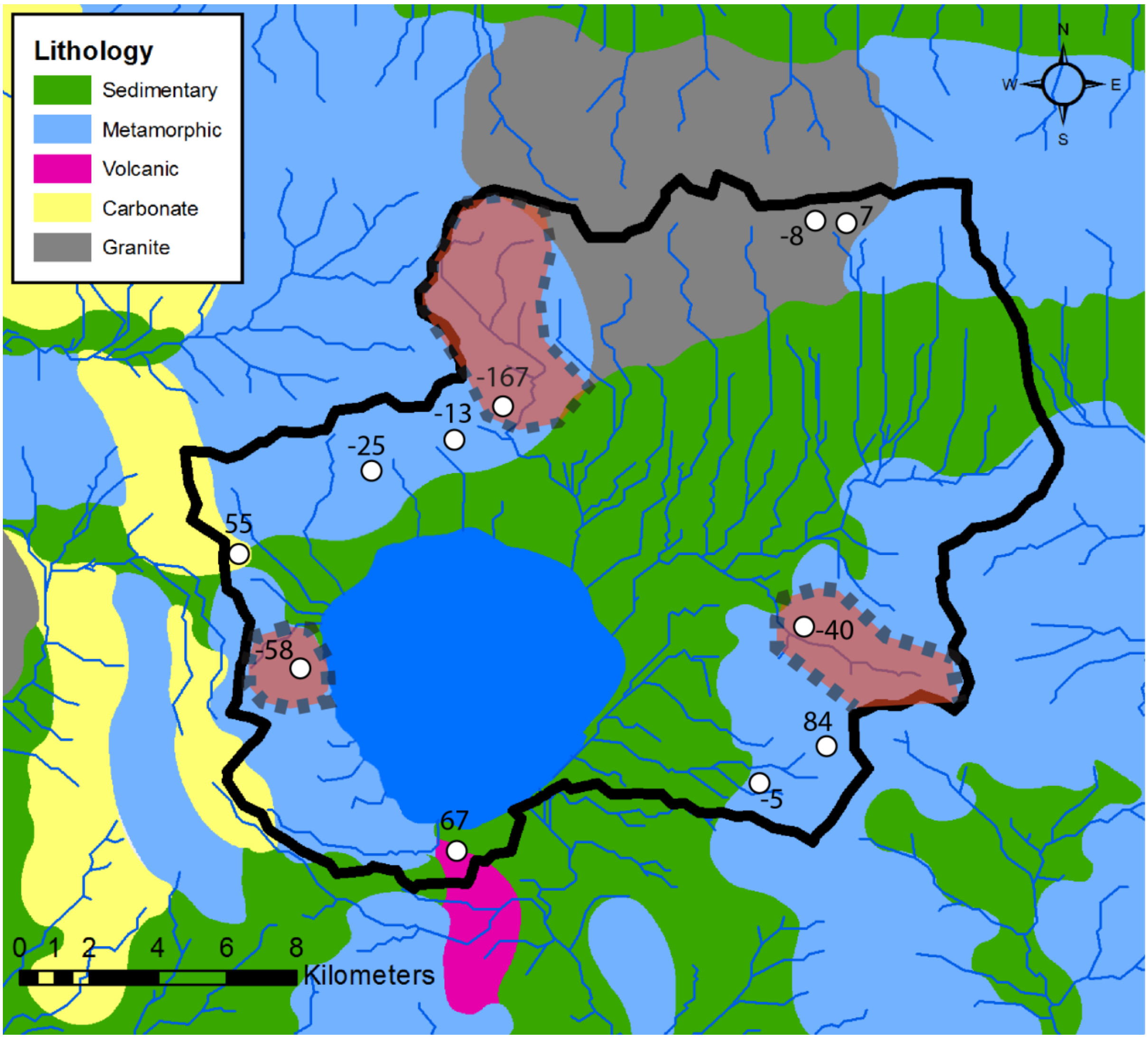

Figure S16: Simplified distribution of geological units for Lake Dojran catchment based on field mapping by A.F. and data from ref.[32,33]. This map was created with ArcGIS Desktop Advanced 10.4 software (https://esriaustralia.com.au/arcgis-desktop). White circles represent locations of bedrock samples. Values are $\delta^{234}U$. Areas highlighted in red with dashed borders represent proposed areas that would need to be preferentially eroded to explain negative $\delta^{234}U$ excursions in the sediment record by a change in sediment provenance solely. External uncertainty for $\delta^{234}U$ is 1.8 ‰.
